# Nucleolar TFIIE plays a role in ribosomal biogenesis and performance

**DOI:** 10.1093/nar/gkab866

**Published:** 2021-09-28

**Authors:** Tamara Phan, Pallab Maity, Christina Ludwig, Lisa Streit, Jens Michaelis, Miltiadis Tsesmelis, Karin Scharffetter-Kochanek, Sebastian Iben

**Affiliations:** Department of Dermatology and Allergic Diseases, Ulm University, Ulm, Baden-Württemberg, 89081 Germany; Department of Dermatology and Allergic Diseases, Ulm University, Ulm, Baden-Württemberg, 89081 Germany; Bavarian Center for Biomolecular Mass Spectrometry, Technical University Munich, Freising, Bavaria 85354, Germany; Institute of Biophysics, Ulm University, Ulm, Baden-Württemberg 89081, Germany; Institute of Biophysics, Ulm University, Ulm, Baden-Württemberg 89081, Germany; Institute of Physiological Chemistry, Ulm University, Ulm, Baden-Württemberg 89081, Germany; Department of Dermatology and Allergic Diseases, Ulm University, Ulm, Baden-Württemberg, 89081 Germany; Department of Dermatology and Allergic Diseases, Ulm University, Ulm, Baden-Württemberg, 89081 Germany

## Abstract

Ribosome biogenesis is a highly energy-demanding process in eukaryotes which requires the concerted action of all three RNA polymerases. In RNA polymerase II transcription, the general transcription factor TFIIH is recruited by TFIIE to the initiation site of protein-coding genes. Distinct mutations in TFIIH and TFIIE give rise to the degenerative disorder trichothiodystrophy (TTD). Here, we uncovered an unexpected role of TFIIE in ribosomal RNA synthesis by RNA polymerase I. With high resolution microscopy we detected TFIIE in the nucleolus where TFIIE binds to actively transcribed rDNA. Mutations in TFIIE affects gene-occupancy of RNA polymerase I, rRNA maturation, ribosomal assembly and performance. In consequence, the elevated translational error rate with imbalanced protein synthesis and turnover results in an increase in heat-sensitive proteins. Collectively, mutations in TFIIE—due to impaired ribosomal biogenesis and translational accuracy—lead to a loss of protein homeostasis (proteostasis) which can partly explain the clinical phenotype in TTD.

## INTRODUCTION

The biogenesis of ribosomes starts with the synthesis of pre-ribosomal RNA (rRNA) by RNA polymerase I. The pre-rRNA is co-transcriptionally matured, processed and assembled with ribosomal proteins (RP) to pre-ribosomal particles within the nucleolus (1). Ribosomal biogenesis requires the concerted action of all three RNA polymerases. While RNA polymerase I and III provide the rRNAs for ribosomal biogenesis (60% of total transcription in growing yeast cells), 50% of RNA polymerase II transcription activity is devoted to the production of mRNAs coding for ribosomal proteins (2). 7500 ribosomal subunits per minute are synthesized in a growing HeLa cell, using up some 300 000 ribosomal proteins (3). All RNA polymerases are dependent on unique sets of basal transcription factors that recognize the core promoter, recruit the RNA polymerases and organize the first steps of productive transcription (4–6). Some of these basal transcription factors are shared between the RNA polymerases like TBP, that decorated with class-specific associated proteins, is essential for all three RNA polymerases (7,8). The DNA-repair factor TFIIH, consisting of ten subunits, is a basal transcription factor of RNA polymerase II. TFIIH melts the promoter and contributes to promoter escape of the polymerase (9). TFIIH is also essential for RNA polymerase I transcription (10) as an elongation factor (11,12). Mutations in TFIIH cause the photosensitive form of the multisystem disorder trichothiodystrophy (TTD), a syndrome with delayed development, microcephaly, neurological degeneration, recurrent infections and eponymous skin and hair abnormalities. TTD is mainly attributed to a transcription syndrome (13,14). However, recently discovered mutations in aminoacyl-tRNA synthetases that result in TTD may shift this classification towards a ‘gene expression syndrome’ referred to a disturbed protein translation at the ribosome (15,16).

TFIIH mutations can also cause the premature aging disease Cockayne syndrome (CS) that resembles TTD with symptoms of developmental delay, microcephaly and neurodegeneration. Earlier TFIIH mutations have been reported to affect RNA polymerase I transcription and the processing of the pre-rRNA in cellular and mouse models of CS and TTD (11,12). Interestingly, a recent study addressed cellular consequences of CS-mutations for RNA polymerase I transcription and uncovered disturbed RNA polymerase I transcription to be responsible for the generation of highly defective ribosomes. In the same report, defective ribosomes were found to display a profoundly reduced translational accuracy giving rise to a loss of proteostasis that in turn represses RNA polymerase I transcription (17). As mutations in subunits of RNA polymerase I or the basal transcription factor UBF were identified in syndromes of childhood neurodegeneration (18–20), it is tempting to speculate that a disturbed RNA polymerase I transcription might be causally involved in the neurodegeneration of CS and TTD.

A subset of TTD cases is caused by mutations in the RNA polymerase II transcription factor TFIIE, respective the β-subunit of this heterodimeric factor (13,21). During the last steps of transcription initiation of RNA polymerase II, TFIIEβ binds to the preinitiation complex (PIC) and recruits TFIIEα and TFIIH to the initiation site. After phosphorylation of RNA polymerase II by TFIIH, TFIIEα is released from the PIC before DNA opening, while TFIIEβ dissociates after DNA opening (14,22).

We here hypothesize that TTD is a syndrome with a pathophysiological involvement of ribosomal biogenesis and protein homeostasis. If this is the case, TFIIEβ should be involved in these central processes as its mutation provokes the same TTD disease.

Here, we discovered an unanticipated role of TFIIE in rRNA synthesis, processing and performance. We detected TFIIE in the nucleolus and showed TFIIE binding to actively transcribed rDNA. TFIIEβ-deficient TTD cells display reduced RNA polymerase I binding to the rDNA and disturbed pre-rRNA maturation. Impaired rRNA maturation subsequently leads to reduced ribosomal stability and unbalanced ribosomal composition in NP-TTD cells. As a consequence, ribosomes of TFIIEβ-deficient TTD cells show increased translational inaccuracy and an imbalanced protein homeostasis

## MATERIALS AND METHODS

### Reagents and resources

Antibodies and oligonucleotides are given in Supplementary Tables S1 and S2.

### Cell culture

Healthy WT controls were grown in DMEM (41965–039, Gibco) supplemented with 10% FBS and 1% penicillin–streptomycin. SV40-immortalized human NP-TTD fibroblasts (TTD218UT, TTD28N) were cultured in DMEM/HAM-F10 (P04-12500, PAN BioTech) in a ratio 1:1 supplemented with 10% FSB and 1% penicillin-streptomycin. Reconstituted cells (218UT TFIIEβ, 28N TFIIEβ) were grown in DMEM/HAM-F10 (1:1) supplemented with 10% FBS, 1% penicillin-streptomycin and 2 μg/ml puromycin. All cells were cultured at 37°C and 5% CO_2_. For RNA polymerase I transcription inhibition, cells on culture dishes with 80% cell density were incubated with 1 μM CX5461 for 4 h.

### Sequence analysis of D187Y variation

The homozygous point mutation in NP-TTD genome was verified by primers binding 100 nucleotides up- and downstream from the variation (forward: 5′ GTATACTGAGTGTGTTAAGGAAATG 3′; reverse 5′ AAAGCTCACCTTCATCCAC 3′). PCR product was used for Sanger-sequencing and data were analyzed by using CLC Workbench 7.7.3.

### Reconstitution of 218UT TFIIEβ and 28N TFIIEβ cell lines

NP-TTD cells were reconstituted with a plasmid overexpressing wild-type TFIIEβ-GFP. 2 × 10^6^ TTD cells were transfected with 3 μg plasmid via electroporation using Neon™ Transfection System (MPK1096, Invitrogen) with following parameters: 1100 V, 20 ms and 2× pulses. Plasmid transfection was verified by flow cytometry. Cells were washed in PBS and flow cytometry analysis were performed in FACS buffer (PBS including 2% FBS). The data was analyzed with FlowJo Software.

### Immunofluorescence

Cells were seeded on pölylysine covered slides for 1 day. 80% confluent cells were washed with PBS, fixed with 4% paraformaldehyde (4°C) for 15 min, washed with PBS, permeabilized with 0.3% Triton X-100/PBS and blocked at RT for 1 h with 5% BSA including 10% goat serum. Antibodies were diluted in Dako Antibody Diluent and cells were incubated with primary antibodies at 4°C overnight in a moist chamber. Cells were washed with PBS, incubated with secondary antibodies at RT for 1 h in a moist chamber, washed with PBS and incubated at RT for 1 h with the DNA probe SPY650-DNA (SC501, SpiroChrome). After washing with PBS, cells were embedded in Dako mounting medium. Images were taken by fluorescence microscope (Zeiss Axio Imager M1, 64 oil objective), confocal microscope (Zeiss Axio Observer Z1 confocal laser scanning microscope, 100 oil objective) and processed and quantified by using ImageJ.

### Super-resolution microscopy

Super-resolution optical microscopy images were taken using a custom built dual-color 3D STED microscope (23). Therefore, 3 × 10^4^ cells were seeded on glass Ibidi μ-slide 8-well glass-bottom plates and incubated o/n. Cells were subsequently fixed with EM-grade PFA and immunostaining was performed as described above. Finally, cells were mounted in 97% 2,2′-thiodiethanol (TDE) for index matching. Antibodies used for immunofluorescence are given in Supplementary Table S1. Typically, an excitation power of 1 μW and a depletion power of 1.3 mW and a repetition rate of 1Mhz were used. The STED images have a pixel size of 20 nm and were captured with a pixel dwell time of 300 μs. Images were analyzed using ImageJ 1.51f and a Gaussian Blur of σ = 1 was applied. For better visualization brightness and contrast, settings were adapted for the images.

### Real-time qPCR standard curve analysis

One microgram of total RNAs were reverse transcribed with Moloney murine leukemia virus Reverse Transcriptase (M170B, Promega). 100 ng cDNA and FastStart Universal SYBR Green Master (04913850001, Roche Diagnostics) were used for real-time qPCR analysis (denaturation at 95°C for 10 s, annealing at 60–68°C for 30 s, elongation at 72°C for 30 s). A prepared standard curve of the oligonucleotide of interest with linear regression with *R*^2^-values >0.8 was used for calculation of the absolute amount (ng) of the oligonucleotide of interest within 100ng total cDNA. Primers used for qPCR analysis are listed in Supplementary Table S2.

### Chromatin immunoprecipitation (ChIP)

Eighty percent confluent cells were fixed with 1% formaldehyde (28908, Thermofisher Scientific) at RT for 10 min and 0.125 M glycine was used to stop cross-linking reaction. Cells were washed with PBS, harvest and lysed with ChIP cell lysis buffer (5 mM HEPES pH 8.0, 85 mM KCl, 0.5% NP-40 (Pierce) and 1:50 cOmplete proteinase inhibitor cocktail mix (Roche)) on ice for 10 min. Pelleted Chromatin was sonicated in ChIP sonicate buffer (50 mM HEPES pH 7.9, 140 M NaCl, 1 mM EDTA, 1% Triton X 100, 0.1% Na-deoxycholate, 0.1% SDS, 0.5 mM PMSF, 1:50 cOmplete) by a Focused-ultrasonicator (Covaris M220, 1 ml) to yield chromatin fragments with an average size of 600 bp. For chromatin precipitation, Protein A agarose beads (20334, Pierce) or Protein A magnetic Dyna beads (10002D, Invitrogen) were used. After incubating 10 μg chromatin with antibodies at 4°C overnight in IP diluent (0.1% SDS, 1% Triton X 100, 1 mM EDTA pH 8.0, 16.7 mM Tris–Cl pH 8.0, 167 mM NaCl, 1:50 cOmplete), chromatin-antibody-complexes were washed first with low salt buffer (0.1% SDS, 1% Triton X 100, 2 mM EDTA, 20 mM Tris–Cl, pH 8.1, 150 mM NaCl), then with high-salt buffer (0.1% SDS, 1% Triton X 100, 2 mM EDTA, 20 mM Tris–Cl, pH 8.1, 500 mM NaCl), with LiCl buffer (10 mM Tris–Cl pH 8.0, 250 mM LiCl, 1% NP40, 1% Na-deoxycholic acid, 1 mM EDTA). and twice with TE buffer (10 mM Tris pH 8, 1mM EDTA). ChIPed DNA was eluted from beads in μChIP elution Buffer (10 mM Tris–Cl pH 8, 1 mM EDTA pH 8, 1% SDS, 1% proteinase K) for 2 h at 60°C with 900 rpm. ChIPed DNA was purified by using QIAquick^®^ Nucleotide Removal Kit (28306, Qiagen) according to the manufacturer's protocol. Samples were qualitative or quantitative analyzed via PCR or qPCR, respectively. For qualitative ChIP analysis, samples were loaded on 1% agarose gel and visualized by using Ged-Doc-It Imager (UVP). For sequential ChIP analysis (Re-ChIP), samples were released after the first round of precipitation by incubation in 10 mM DTT for 30 min at 37°C at 500 rpm and incubated with the respective other antibody for the second round of precipitation. Primers and antibodies used for ChIP analysis are given in Supplementary Tables S1 and S2.

### Nuclear Run-on Assay

5 × 10^7^ cells were grown on 15 cm culture dishes until 80% cell density, washed with cold PBS and collected in a 50 ml falcon tube by scraping and centrifuging for 5 min 1200 rpm. Cells were subsequently lysed with 4 ml NP-40 lysis buffer (10 mM Tris–HCl pH 7.4, 10 mM NaCl, 3 mM MgCl_2_, 0.5% NP-40) on ice for 5 min and centrifuged at 1000 rpm for 10 min. Pellets were resuspended in 200 μl nuclear freezing buffer (50 mM Tris–HCl pH 8.3, 40% v/v glycerol, 5 mM MgCl_2_, 0.1 mM EDTA) and incubated with 50 μl of 5× Run-on buffer (25 mM Tris–HCl pH 8.0, 25 mM MgCl_2_, 500 mM KCl, 1.25 mM ATP, 1.25 mM GTP, 1.25 mM CTP) and 1.25 mM Biotin-labeled UTP (AM8452, Invitrogen) for 40 min at 37°C with 300 rpm shaking. Total RNA was extracted by using RNeasy Kit (74004, Qiagen) according to the manufactory's protocol. Freshly transcribed RNAs with Biotin-labeled UTP were isolated from total RNA by using Dynabeads™ MyOne™Streptavidin C1 (65001, Invitrogen) according to the manufactory's protocol. In short, 15 μl beads per reaction were washed with Solution A (DEPC-treated 0.1 M NaOH, DEPC-treated 0.05 M NaCl), subsequently with Solution B (0.1 M NaCl) and finally with Binding and Washing Buffer (10 mM Tris–HCl pH 7.5, 1 mM EDTA, 2M NaCl, 1:50 RNasin^®^ (N2511, Promega)). Prepared Dyna beads were incubated with total RNA for 10 min at RT, washed twice with Binding and Washing Buffer. Isolated RNA was directly reversed transcribed with Moloney murine leukemia virus Reverse Transcriptase (M170B, Promega) followed up with qPCR analysis. Primers used for Nuclear Run-on Assay are listed in Supplementary Table S2.

### Western blot analysis

Cells were grown on 15 cm culture dishes until 80% cell density, harvest and lysed with 100 μl lysis buffer (10% glycine, 1% Triton X 100, 137 mM NaCl, 20 mM Tris pH 8, 2 mM EDTA pH 8.1). 100 – 300 μg protein was loaded on 10–12% SDS-PAGE and transferred at 4°C overnight to a nitrocellulose blotting membrane (A29434119, GE Healthcare) in transfer buffer (25 mM Tris pH 8, 192 mM glycine, 5% methanol). Membranes were blocked at RT for 1 h with 5% milk powder and 0.1% Tween 20 (diluted in PBS), washed in PBS, incubated with primary antibodies at 4°C overnight, washed with PBS and incubated with secondary antibodies at RT for 1 h. Membranes were developed using Fusion Fx7 (Vilber). Images were processed and quantified by using ImageJ. Antibodies used for western blot analysis are listed in Supplementary Table S1.

### Northern Blot analysis

Five microgram total RNA were denaturated at 65°C for 15 min in RNA loading dye (50% formamide, 7.5% formaldehyde, 1× MOPS, 0.5% ethidium bromide) and immediately placed on ice for 5 min. Samples were separated on a 0.9% agarose gel (diluted in 1× MOPS) with 80V for 2.5–3 h. The gel was soaked in 20× SSC (3M NaCl, 0.3 M Na citrate, adjust to pH 7) and RNAs were transferred to Amersham Hybond membrane (RPN303S, GE Healthcare) soaked in 2× SSC. Membrane was pre-hybridized for 2 h at 60°C with pre-hybridization buffer (50% formamide, 0.1% SDS, 8× Denhards solution, 5x SSC buffer, 50 mM NaP buffer, 0.5 mg/ml t-RNA). Probes were synthesized by T4 polynucleotide kinase using radioactive [^32^P] γATP. Membranes were hybridized with radioactive ^32^P-labeled oligonucleotides using pre-hybridization buffer at 60°C for 1 h and subsequently at 37°C overnight. Finally, membranes were exposed to X-ray film and quantified with ImageJ after Wang and Pestov (24). For rRNA processing pathway analysis we used probes binding to the region ITS1 (5′ GGGCCTCGCCCTCCGGGCTCCGTTAATGATC 3′) and ITS2 on the rDNA (5′ CTGCGAGGGAACCCCCAGCCGCGCA 3′).

### Ribosome preparation

Ribosome preparation was performed after Penzo *et al.* (25). 80% confluent cells were washed, harvest and lysed with cell lysis buffer (10 mM Tris–HCl pH 7.5, 10 mM NaCl, 3 mM MgCl_2_, 0.5% Nonidet P40) on ice for 10 min. Samples were centrifuged for 12 min at 16 900 × g and lysates were incubated with protein synthesis master mix (150 mM HEPES–KOH pH 7.5, 400 mM KCl, 250 μM l-amino acid mixture, 1.25 mM GTP, 10 mM ATP, 25 mM creatine phosphate, 2 mM spermidine, 2.5 mM DTT, 5mM magnesium acetate, 0.9 mg/ml creatine kinase) for 10 min at 37°C. Subsequently, samples were transferred to a sucrose gradient, consisting of a bottom sucrose layer (30 mM HEPES–KOH pH 7.5, 70 mM KCl, 2 mM magnesium acetate, 1 mM DTT, 1 M sucrose) and a high-salt-top layer (30 mM HEPES KOH pH 7.5, 0.5 M KCl, 2 mM magnesium acetate, 1 mM DTT, 0.7 M sucrose). After ultracentrifugation (Optima™ MAX-XP Ultracentrifuge, Beckman Coulter) for 15 h at 4°C with 110 000 × g, pelleted ribosomes were resuspended in ribosome solution (10 mM Tris–HCl pH 7.5, 2mM magnesium acetate, 100 mM ammonium acetate).

### Ribosome stability assay using BisANS

Five microgram isolated ribosomes were treated with 2 M urea for 2 h. Conformational changes of proteins were detected by the probe BisANS (4,4′-dianilino-1,1′-binaphthyl-5,5′-disulfonic acid, dipotassium salt). BisANS is nonfluorescent in water and only becomes appreciably fluorescent when bound to the hydrophobic site of proteins. Hence, BisANS is a sensitive indicator of protein unfolding. Fluorescent was detected by a plate reader Varioskan™ LUX (Thermofisher Scientific) using excitation 375 nm/emission 500 nm.

### Mass spectrometry analysis

Mass spectrometry analysis of isolated ribosomes from NP-TTD cell line TTD218UT and reconstituted cell line 218UT TFIIEβ were performed by the Bavarian Center for Biomolecular Mass Spectrometry, Technical University Munich (Germany). Label-free quantification of mass spectrometry data was analyzed using Excel and the program software R Studio.

### Fluorescence *in situ* hybridization

Fluorescence *in situ* hybridization was performed after (26). In short, cells were seeded on polylysine covered slides and let grow until 80% cell density. After fixation with 4% paraformaldehyde (4°C) for 12 min at RT, cells were washed twice with PBS and sequentially permeabilized with 0.3% Triton X-100/PBS for 10 min at RT. Cells were washed twice with washing buffer (2× SSC, 10% formamide) and hybridization was performed in hybridization buffer (10% formamide, 2× SSC, 0.5 μg/μl tRNA, 10% dextran sulfate, 50 μg/ml BSA, 10 mM ribonucleoside vanadyl complexes, 0.5 μg/μl probe) for 3 h at 37°C. After washing cells twice with washing buffer and once with PBS, cells were incubated in DAPI solution (0.1 μg/ml) for 5 min at RT. Finally, cells were washed twice in PBS and mounted. Images were taken with a Compact Fluorescence Microscope (BZ-X800E, Keyence). The CTCF (corrected total cell fluorescence) in the nucleolus (ITS1 probe) or the nucleus (U2) were analyzed in at least 100 cells per cell line by using ImageJ and experiments were performed at least twice per cell line. For Fluorescence in situ hybridization we used probes binding to ITS1 (CCT*CGCCCTCCGGGCT*CCGTTAATGAT*C) and U2 (A*AACTGATAAGAACAGATACT*ACACTTGATCTTAGCCAAA*A). * represents the nucleotide conjugated to Cy3 in ITS1 probe or Cy5 in U2 probe.

### Polysomal profiling

5 × 107 cells were treated with 100 μg/ml Cycloheximide (C7698-1G, Sigma) for 30 min at 37°C. Cells were collected by scraping to 50 ml falcon tubes and centrifuging for 5 min 1200 rpm. Pelleted cells were mechanically disrupted with a Dounce homogenizer in 1.5× Dignam A (incl. 1:1000 DTT, 1:50 cOmplete proteinase inhibitor cocktail mix (Roche), 100 μg/ml Cycloheximide). Samples were rested on ice for 1 min and subsequently centrifuge for 10 min at 1000 × g at 4°C. The supernatant was collected and centrifuged twice for 5 min at 12 000 × g at 4°C. The new supernatant was considered as the cytoextract and 500 μg cytoextract was loaded on a 10–50% sucrose gradient. After ultracentrifugation (OptimaTM MAX-XP Ultracentrifuge, Beckman Coulter) for 3 h at 4°C with 100 000 × g, the gradient was analyzed at OD_260 nm_. The linear sucrose gradient as well as the measurements at OD_260 nm_ were obtained by Piston Gradient Fractionator™ (BioComp).

### Luciferase-based translation fidelity assay with plasmid transfection

The translation fidelity assay via plasmid transfection includes the control reporter luciferase *Renilla* and experimental reporters *Firefly* (PC, MUT). *Renilla* and *Firefly* were expressed on separate plasmids (kindly provided by Vera Gorbunova). Cells were cultured in 10 cm culture dishes until 80% cell density. 15 × 10^4^ cells were harvest and co-transfected with 0.1 μg control plasmid *Renilla* and 5μg reporter plasmid *Firefly* (Neg or Mut) via electroporation using Neon™ Transfection System (MPK1096, Invitrogen) with following parameters: 1100 V, 20 ms and 2× pulses. Subsequently, cells were seeded in 5 × 10^4^ cells/well in white 96-well plates and were grown 24 h. Luciferase activities were detected by using Dual-Glo^®^ Luciferase Assay System (E2920, Promega) according to the manufactory's protocol.

### Luciferase-based translation fidelity assay with mRNA transfection

The translation fidelity assay via mRNA transfection includes the control reporter luciferase *Renilla* and experimental reporters *Firefly* (PC or MUT). *Renilla* and *Firefly* were expressed on one plasmid (kindly provided by Markus Schosserer). Plasmids were transcribed to mRNAs by using Ampli Cap-Max T7 High Yield Message Marker Kit (C-ACM04037, CellScript) according to the manufactory's protocol. 10^5^ cells/well in 100 μl culture medium were seeded in white 96-well plate and were grown overnight. 500ng mRNA/well in 50 μl OptiMEM (31985070, Gibco) and 1 μl/well of Lipofectamine^®^ MessengerMAX mRNA Transfection Reagent (LMRNA003, Invitrogen) in 50 μl OptiMEM were incubated for 10 min at RT. mRNA dilution and Lipofectamine dilution were mixed and incubated for further 15 min at RT. After removing the old media from cells, 100 μl of mRNA-Lipofectamine-OptiMEM mixture were transferred to each well and cells were grown for 24 h. Luciferase activities were detected by using Dual-Glo^®^ Luciferase Assay System (E2920, Promega) according to the manufactory's protocol.

### Protein synthesis analysis

Protein synthesis was detected by using Protein Synthesis Assay Kit (601100, CaymanChemical) according to the manufactory's protocol. Briefly, 2.5 × 10^4^ cells/ml were grown in white 96-well plate overnight, incubated with OPP (O-Propargyl-puromycin) working solution for 1 h, fixed, and stained with 5 FAM-Azide solution for 30 min. OPP binds to newly synthesized polypeptides and 5 FAM-azide is fluorescent upon binding to OPP. Fluorescence was detected by using a plate reader Varioskan™ LUX (Thermofisher Scientific) using excitation 485 nm / emission 535 nm.

### ^35^S Methionine labeling

Protein translation was analyzed by using metabolic labeling of cells with ^35^S methionine. Therefore, 5 × 10^4^ cells per reaction were harvest in 1.5 ml reaction tube by centrifuging for 5 min at 3000 rpm, and incubated at 37°C for 30 min in 100 μl methionine-free DMEM (21013-024, Gibco) supplemented with 10% FBS and 1% penicillin–streptomycin. Subsequently, 10 μl culture medium with 1% ^35^S-labeled methionine was added to cells and cells were incubated for further 1 h. After centrifuging cells for 5 min at 3000 rpm, cell pellet was resuspended in 100 μl 1mg/ml BSA and each reaction was vortex for 3 s. 1 ml of cold 10% TCA was added, vortexed for 3 s and reactions were incubated on ice for 1 h. Reaction was transferred onto glass fiber prefilters (Merck, APFC02500) and washed with cold 10% TCA and subsequently with cold 70% ethanol. Glass fiber prefilters were air dried for 5 min and the amount of ^35^S-labeled methionine was analyzed with 5 ml liquid scintillation cocktail Ultima Gold™ (6013329, PerkinElmer) by Liquid Scintillation Analyzer Tri-Carb 4910 TR (PerkinElmer).

### 20S Proteasome activity analysis

20S Proteasome activity was determined by using 20S Proteasome Assay Kit (10008041, CaymanChemical) according to the manufactory's protocol. In short, 5 × 10^5^ cells/well in 100 μl culture medium were seeded in white 96-well plate, lysed, and incubated with a 20S specific substrate solution for 1 h. The 20S specific substrate is fluorescent upon cleavage and fluorescence was detected by using a plate reader Varioskan™ LUX (Thermofisher Scientific) using excitation 360 nm / emission 480 nm.

### Heat sensitivity analysis

Heat sensitivity analysis was adapted from Treaster and colleagues (27). Cells were grown on 15 cm culture dished to 80% cell density. 4 × 10^6^ cells were harvest and its PCV (packed cell volume) was estimated. Cells were dissolved in 1.5× PCV of Dignam A buffer (10 mM KCl, 10 mM Tris pH 7.9, 1.5 mM MgCl_2_, 1:50 cOmplete proteinase inhibitor mix) for 15 min on ice. Cells were lysed by passing cells 50 times through a 23G syringe. Cytoplasmic extract was separated from nuclei and cell debris by centrifugation for 5 min at 4000 rpm at 4°C and subsequently by ultracentrifuge for 1 h with 100 000 × g at 4°C. 150μg of protein lysate was heat-treated at 99ºC for 15 min and subsequently centrifuged for 5 min with 14 000 rpm at 4°C. The pellet was resuspended in 10 μl 4M urea and the protein concentration of both pellet and supernatant was determined by using the method of Bradford (5000006, Bio-Rad). Finally, the percentage of pelleted protein in relation to total protein was calculated.

### Growth kinetic

2 × 10^5^ cells were seeded per well in a 6-well culture plate. For each time point 3, wells were detached using 1x trypsin and manually counted using a hemocytometer (Neubauer chamber, 0.100 mm depth, 0.0025 mm^2^ area). Cells were counted every 48 h over a period of 10 days.

### Statistical analysis

All plots were created by using GraphPad Prism version 7.0.3, Excel Microsoft Office Professional Plus 2013 or R version 3.4.4. Differences between means ± standard deviation (SD) of at least three independent experiments were assessed with ONE Way ANOVA when comparing samples, and with two-way ANOVA followed by Sidak post-hoc analysis, when comparing samples in groups, unless differently described. ns *P* > 0.05, * *P* ≤ 0.05, ** *P* ≤ 0.01, *** *P* ≤ 0.001, **** *P* ≤ 0.0001.

## RESULTS

### TFIIE is enriched in transcriptionally active nucleoli and binds to ribosomal DNA

**Figure 1.**
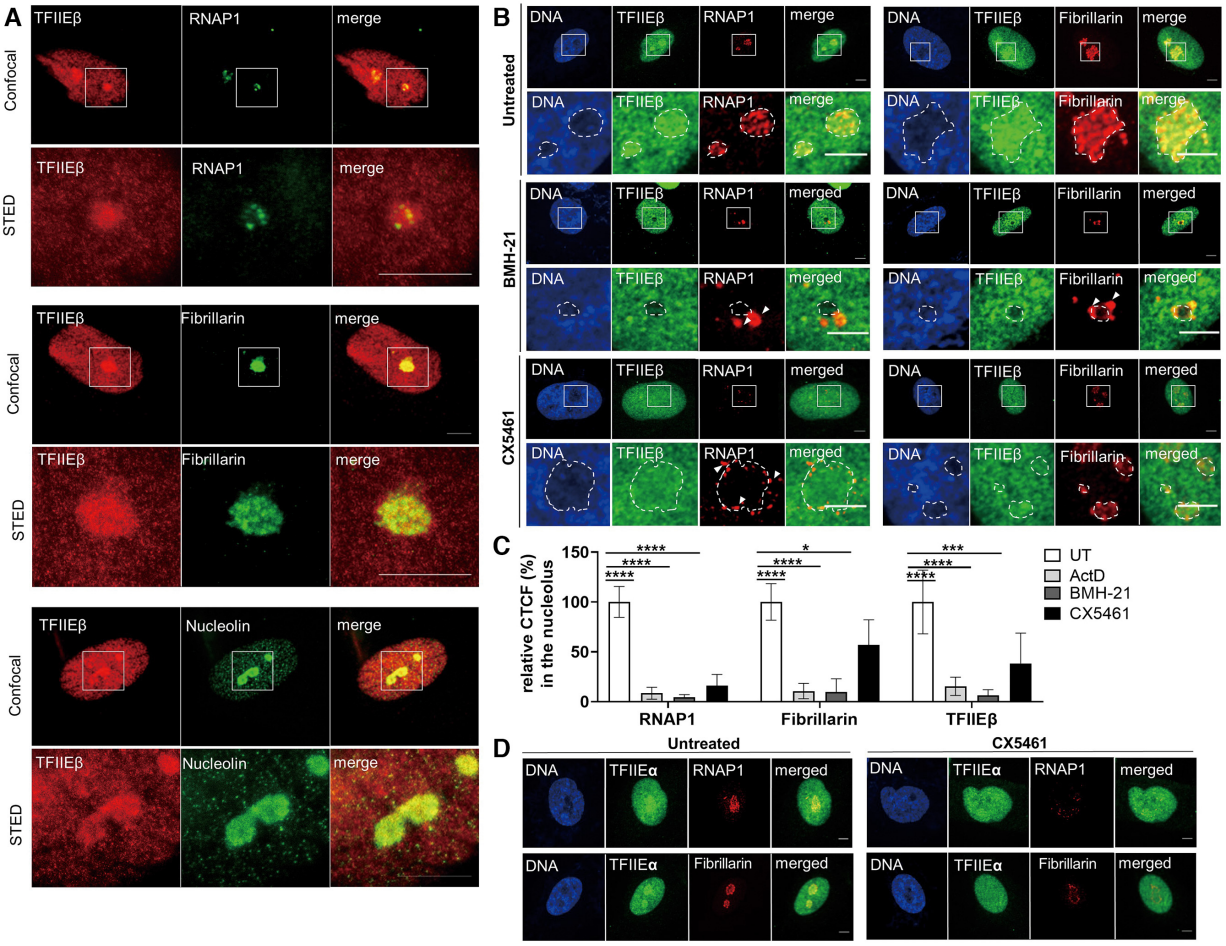
Both TFIIE subunits localize to the nucleolus. (**A**) Super resolution STED microscopy images showing co-localization of TFIIEβ with nucleolus markers RNA polymerase I, Fibrillarin and Nucleolin. Scale bar 5 μm. More STED images of TFIIH (as a positive control), TFIIF and TFIIEβ after specific inhibition of RNA polymerase I transcription by BMH-21 are given in the Supplement S1A-B. Analysis of STED images are given in the Supplement S2A–C. (**B**) Confocal immunofluorescence microscopy (100×) of WT cells indicates co-localization of TFIIEβ with RNAP1 and Fibrillarin. Inhibition of RNA polymerase I transcription in WT cells by 1 μM BMH-21 or 1 μM CX5461 result in a re-distribution of both TFIIE from the nucleolar enrichment and an organization of RNA polymerase I to nucleolar caps (arrows). Scale bar 5 μm. More images of TFIIEβ localization after Actinomycin D treatment are given in the Supplement S1C. (**C**) Quantification of CTCF (corrected total cell fluorescence) of IF performed in (B) indicating reduced TFIIEβ staining in the nucleolus after inhibition of RNA polymerase I transcription by Actinomycin D (ActD), BMH-21 or CX5461. (**D**) Confocal immunofluorescence microscopy (100X) of WT cells untreated and treated with CX5461 indicating co-localization of TFIIEα with RNA polymerase I and Fibrillarin. Inhibition of RNA polymerase I transcription results in a non-significant re-distribution of TFIIEα from the nucleolar enrichment. Scale bar 5 μm. Quantification of co-localization studies of TFIIEα upon treatment with CX5461 are given in the Supplement S2D. Data are represented as mean ± SD of at least three independent experiments. Measures were performed in at least 50 cells. * *P* ≤ 0.05, *** *P* ≤ 0.001, **** *P* ≤ 0.0001.

**Figure 2.**
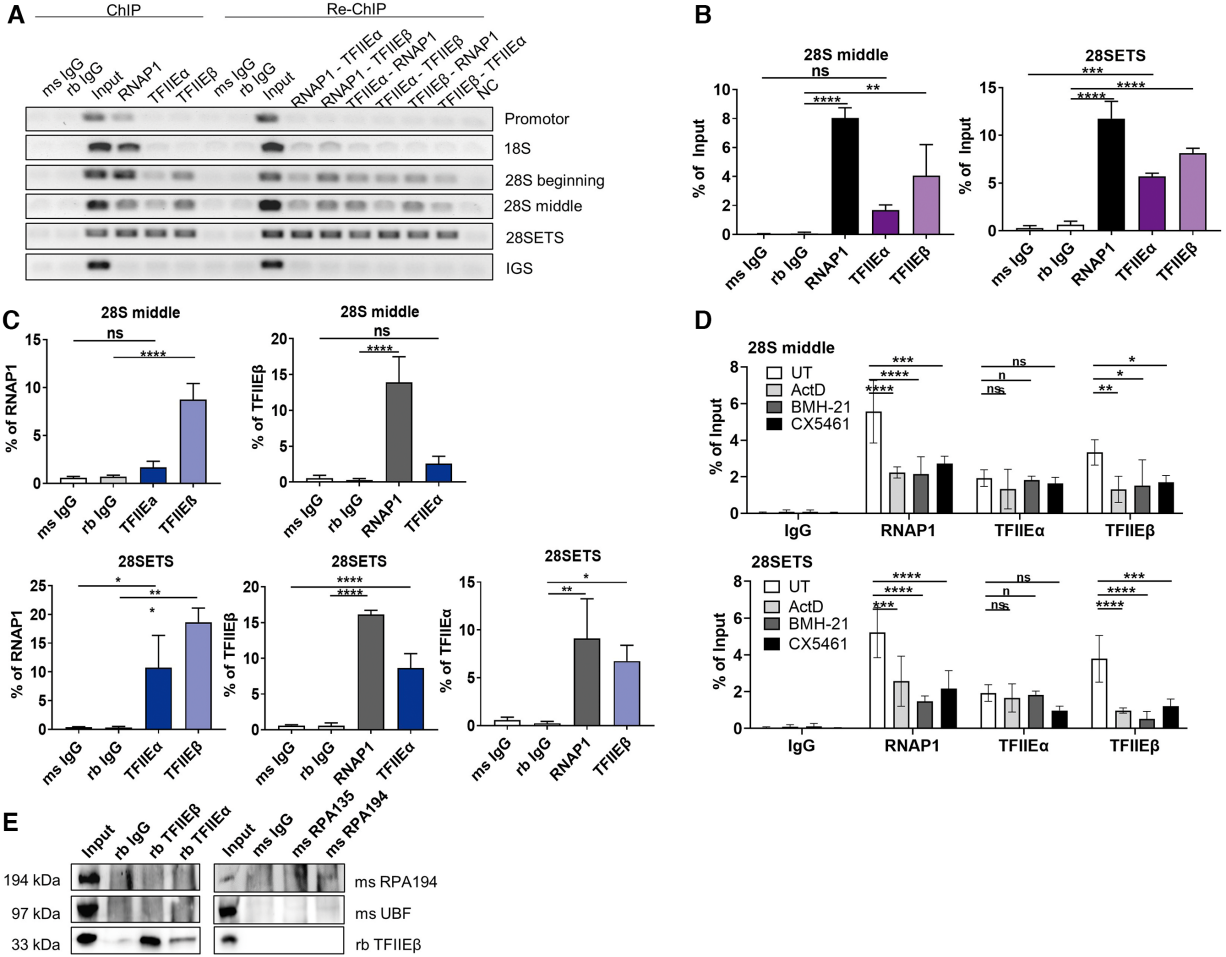
TFIIE associate to active rDNA genes. (**A**) Semi-quantitative PCR analysis of ChIP and sequential ChIP (Re-ChIP) of TFIIE subunits and RNA polymerase I shows no binding of TFIIEβ to the beginning and middle of the rDNA (Promotor, 18S), but to the 28S rDNA coding region (28S beginning - 28SETS). Re-ChIP semi-quantitative analysis shows binding of TFIIE to the same rDNA molecules as RNAP1. Semi-quantitative PCR analysis of the ribosomal intergenic spacer (IGS) region serves as a negative control. (**B**) Quantitative qPCR analysis of ChIP performed in (A) shows significant binding of TFIIEβ to the whole 28S rDNA coding region, while TFIIEα only associates to the end of the 28S rDNA coding region (28SETS). More quantitative qPCR analysis of ChIP at other regions of the rDNA are given in the Supplement S3A. Semi-quantitative and quantitative analysis of ChIP of additionally UBF are given in the Supplement S3B-D. (**C**) Quantitative qPCR analysis of sequential ChIP (Re-ChIP) performed indicates significant binding of TFIIE subunits to the same rDNA molecule as RNA polymerase I. (**D**) Quantification of ChIP analysis showing reduced TFIIEβ and RNA polymerase I binding to the 28S coding region (28S middle, 28SETS after inhibition of RNA polymerase I transcription by Actinomycin D (ActD), BMH-21 or CX5461. Data are represented as mean ± SD of at least three independent experiments. ns *P* > 0.05, *P* * *P* ≤ 0.05, ** *P* ≤ 0.01, *** *P* ≤ 0.001, **** *P* ≤ 0.0001. ms and rb IgG are mouse and rabbit IgG controls, respectively. **(E)** Co-Immunoprecipitation of TFIIE subunits (TFIIEα, TFIIEβ) and RNA polymerase I subunits (RPA194, RPA135) showing direct interaction of TFIIEβ and TFIIEα, RPA194 and RPA135, but no direct interaction between TFIIE subunits with RNA polymerase I subunits.

TFIIE mutations cause TTD, a disease mainly provoked by mutations in the multifunctional TFIIH complex. As the TFIIH complex plays an essential role in RNA polymerase I transcription (10), we have addressed the question if TFIIE is also involved in ribosomal biogenesis. RNA polymerase I transcription takes place in the nucleolus, the densest cellular organelle. To investigate if TFIIE localizes to the nucleolus, co-localization studies employing confocal microscopy with nucleolar proteins were performed and further refined by super resolution STED microscopy (Figure 1A). We observed a distinct enrichment of TFIIEβ in the nucleoli and a close co-localization with RNA polymerase I, Fibrillarin and Nucleolin. Remarkably, this enrichment is lost when RNA polymerase I transcription is blocked by the specific inhibitors BMH 21, CX5461 and Actinomycin D leading to a re-organization of RNA polymerase I in nucleolar caps (Figure 1B-C, Supplementary Supplement S1B, C, Supplement S2A, C) The large subunit of TFIIE, TFIIEα showed a comparable distribution when co-stained with RNA polymerase I (Figure 1D, Supplement S2D left panel). This data indicates that both subunits of TFIIE are enriched in the nucleoli. TFIIH, known to be involved in RNA polymerase I transcription, can also be traced in the nucleolus (Supplement S1A, SupplementS2B left panel). Interestingly, TFIIF, a general transcription factor of RNA polymerase II and one interaction partner of TFIIE, is not enriched in the nucleolus (Supplement S1A, SupplementS2B right panel), indicating an RNA polymerase II- independent localization of TFIIE in the nucleolus. As the TFIIE complex binds to the core promoter DNA of RNA polymerase II genes (14,22) we further explored a potential binding of TFIIE to the ribosomal DNA using ChIP experiments. In fact, binding of TFIIE to the rDNA at the end of the coding region is distinctly traceable, while TFIIE did not bind to the promoter of the rDNA. A specific binding of both subunits of TFIIE to the rDNA is clearly detectable by semi-quantitative and quantitative PCR (Figures 2A, left side, Figure 2B, Supplement S3A). These findings are supported by further experiments depicted in Figure 2A, right panels and Figure 2C. In fact, using ChIP-re-ChIP experiments we show that both TFIIE subunits were precipitated with RNA polymerase I antibodies and antibodies against the basal RNA polymerase I transcription factor UBF (Supplement S3B–D). These data imply that TFIIE, RNA polymerase I and UBF bind to the same rDNA molecules constituting the transcriptionally active fraction of the rDNA. This rDNA binding is significantly reduced for TFIIEβ and RNA polymerase I, when treating the cells with Actinomycin D, BMH21 and CX5461 (Figure 2D). However, a direct interaction of TFIIE with RNA polymerase I could not be observed in co-immunoprecipitation assay (Figure 2E, Supplement S3E). Thus, TFIIE localizes to the nucleolus and associates with transcriptional active rDNA molecules providing evidence for a possible role of TFIIE in ribosomal biogenesis.

### Mutations in TFIIEβ affect rDNA transcription and processing

The two fibroblasts cell lines TTD218UT and TTD28N used in this study were isolated from two different TTD patients bearing the same homozygote mutation in D187Y (Supplement S4A, (13,21)). Both patient cell lines were reconstituted with a wild-type TFIIEβ fused to GFP: 218UT TFIIEβ and 28N TFIIEβ. For the following experiments the patient cell lines were compared to a healthy control (WT) and the respective reconstituted cell line.

As previously described (13), employing western blot analysis we confirmed reduced protein concentration of TFIIEβ in both TTD cell lines indicating that the mutation affects TFIIEβ protein stability (Supplement S4C, D). By contrast, protein levels of TFIIH subunits are stable (Supplement S4C, E). As reported, the abundance of TFIIEα protein is diminished although there are no mutations. These findings were confirmed by immunofluorescence staining of both TFIIE subunits in TTD cells compared to reconstituted cell and WT cell lines (Supplement S4B).

**Figure 3.**
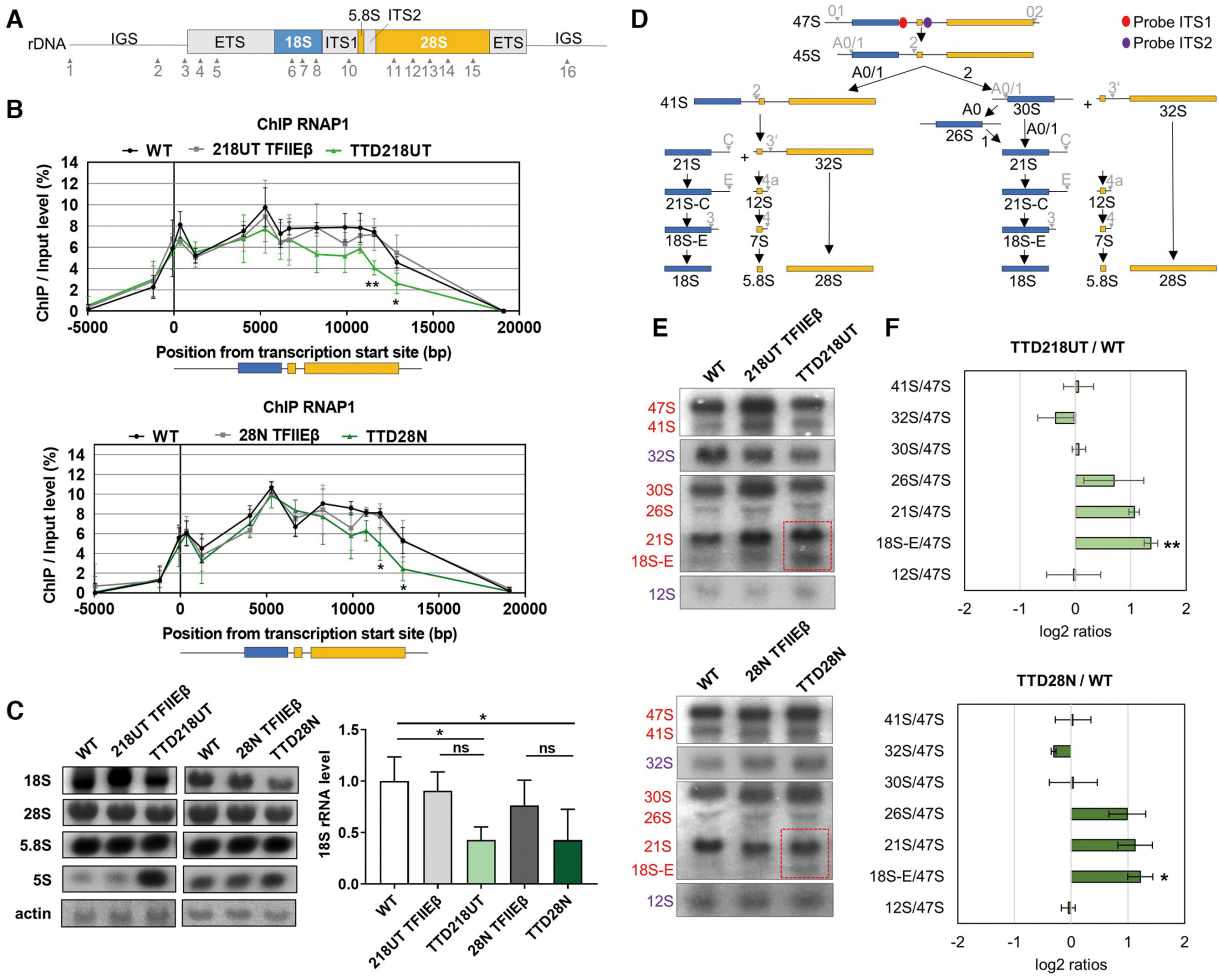
TFIIE influences RNA polymerase I gene occupancy and rRNA processing. (**A**) Schema of the human rDNA unit. The non-coding region ETS, ITS1 and ITS2 (gray) and coding regions 18S (blue), 5.8S and 28S (both in orange) are illustrated. Primers used for ChIP analysis are indicated by arrowheads and numbers. Exact primer sequences are given in Table S2. (**B**) Quantitative qPCR ChIP analysis of RNAP1 in WT, reconstituted and TTD cells indicates reduced binding of RNAP1 to the end of the 28S rRNA coding rDNA (region 14 and 15) in TTD cells. (**C**) Left: Northern Blot analysis of mature 18S, 28S, 5.8S and 5S in WT, reconstituted and TTD cells. Right: Quantification of mature rRNA relative to actin shows decreased mature 18S level in TTD cells compared to WT cells. Quantification of 47S pre-rRNA, 28S, 5.8S and 5S mature rRNA are given in the Supplement S5B. (**D**) Schema of the rRNA processing pathway in human cells; adapted from (45). ITS1 and ITS2 probes positions are marked in red and purple, respectively. (**E**) Northern Blot of WT, reconstituted and TTD cells show accumulation of 21S and 18S-E levels in TTD cells. Red and purple color code indicates rRNA species detected by ITS1 and ITS2 probe, respectively. Full images of the probed membrane are given in the Supplement S5B. (**F**) Analysis of the northern blots in (E) are displayed as Ratio Analysis of Multiple Precursors (RAMP) profiles;(24). Data are represented as mean ± SD of at least three independent experiments. ns *P* > 0.05, *P* * *P* ≤ 0.05, ** *P* ≤ 0.01.

To investigate if the reduced TFIIE levels in TTD cells may impact on RNA polymerase I transcription, we examined gene occupancy of the rDNA by RNA polymerase I in more detail. ChIP experiments were used for the analysis of 16 rDNA regions (Figure 3A) by qPCR to map the density of RNA polymerase I on the ribosomal DNA template and to create a binding profile. The binding profile was then compared between wild-type, reconstituted and TFIIEβ mutated cell lines. As depicted in Figure 3B, the rDNA binding profile of RNA polymerase I is significantly altered in both TTD patient cell lines, with a significant reduction of rDNA binding by RNA polymerase I throughout the entire 28S rDNA. Nuclear Run-on analysis revealed a reduced level of promotor-distal pre-rRNA synthesis. This finding is in line with the reduced gene-occupancy of RNA polymerase I (Supplement S5A). These results imply that the abundance of TFIIE on the rDNA is essential for the proper association with the template and elongation activity of the polymerase.

Since TFIIEβ mutations in TTD cells reduce the binding of RNA polymerase I to the end of the rDNA, we further assessed 47S pre-rRNA levels and mature rRNA abundance by Northern blot analysis. No significant reduction of 47S pre-rRNA was detected (Supplement S5B), however, the amount of mature 18S rRNA was significantly reduced in TTD cells (Figure 3C). The pre-rRNA precursor is co-transcriptionally processed, matured and assembled in pre-ribosomal particles (1). Therefore, and stimulated by previous work (12), we were interested to monitor the processing steps from the pre-rRNA to the mature product by Northern blot analysis. In Figure 3D, the processing steps are schematically depicted and the position of the probes used to perform Northern blots are indicated. This technique allows to visualize and quantitate all processing intermediates within the rRNA maturation pathway. Northern blot analysis and ratio analysis of multiple precursors (RAMP) profile quantification (24) revealed an increased tendency to the accumulation of 21S and a significant accumulation of 18S-E processing intermediate in TTD cells as opposed to WT and reconstituted cells (Figure 3E, F, Supplement S6A, B).

Taken together these results show, that mutation in TFIIEβ weakens the interaction of RNA polymerase I with the template that is followed by the accumulation of processing intermediates.

### TFIIEβ mutation affects ribosomal stability and performance

**Figure 4.**
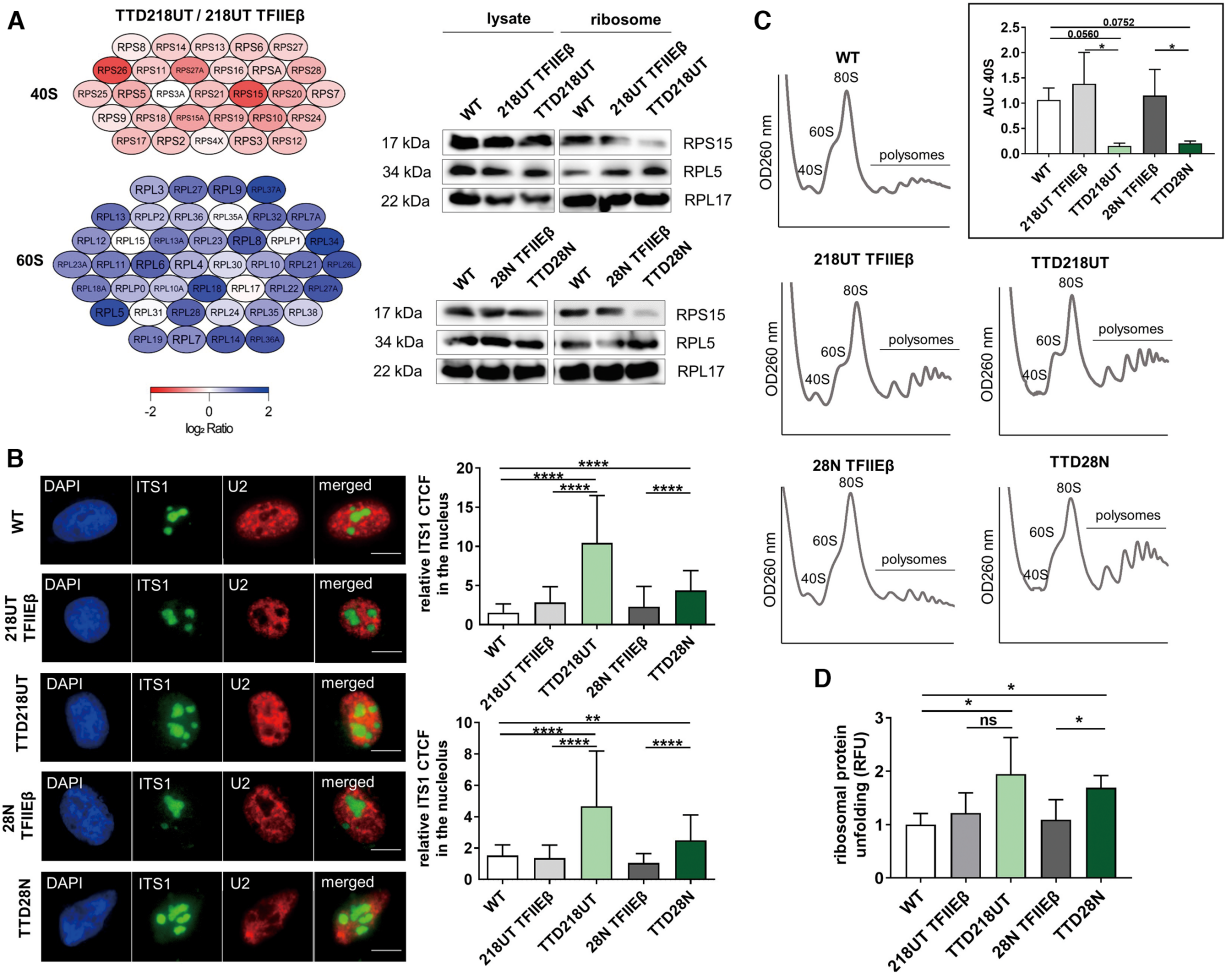
Mutation in TFIIE disturbs ribosomal composition and performance. (**A**) Left: Mass spectrometry analysis shows a non-significant decreased amount of ribosomal protein of the small 40S ribosome subunit and a non-significant increased amount of ribosome protein of the large 60S ribosome subunit in TTD cells compared to reconstituted cells. Ribosomal proteins are color-coded with low detection (red), normal detection (white) and high detection (blue). A volcano plot is given in the Supplement S6C. Right: Western blot analysis of whole cell lysate and isolated ribosomes in all cells showing normal level of ribosomal proteins in lysate, but decreased level of RPS15 and increased level of RPL5 in isolated ribosomes of TTD cells. More western blot analyses of more ribosomal proteins and quantification are given in the Supplement S6D and E. (**B**) Fluorescence in situ hybridization with ITS1 Cy3-labeled probe indicates 18S-E accumulation in the nucleus and nucleolus in TTD cells compared to reconstituted and healthy controls. U2 C5-labeled probe was used as control and indicates no significant differences in TTD cells (Supplement S6F). Quantification of the CTCF was performed as described in materials and methods. Measures were performed in at least 100 cells. (**C**) Polysome analysis shows a strong reduction of free 40S subunits in TTD cells compared to reconstituted and healthy controls. Insert: Quantification of area under the curve (AUC) of 40S peaks in WT, reconstituted and TTD cells indicating reduced AUC of 40S subunit in TTD cells. **(D)** Isolated ribosomes of WT, reconstituted and TTD cells were treated with 2M urea for 2 hrs. Exposed hydrophobic chains of unfolded proteins were quantified by Bis-ANS fluorescence. TTD cells show a higher amount of unfolded ribosomal proteins after partial denaturation. Data are represented as mean ± SD of at least three independent experiments. ns *P* > 0.05, * *P* ≤ 0.05, *** *P* ≤ 0.001, **** *P* ≤ 0.0001.

Next, we asked the question if and how disturbed ribosomal biogenesis affects ribosomal composition and the translation process. While mature 18S rRNA is the structural and functional backbone of the small ribosomal subunit 40S, the 5.8S, 28S rRNA and 5S (a product of RNA polymerase III) are components of the large ribosomal subunit 60S. Since our TTD cells show reduced mature 18S rRNA abundance due to disturbances in rRNA processing, we isolated ribosomes from reconstituted and TTD cells after sucrose gradient centrifugation (25) and performed quantitative analysis of the ribosomal composition by mass spectrometry (Figure 4A, left panel). Although not reaching significance, all detectable ribosomal proteins of the small subunit 40S are reduced in TTD ribosomes compared to the reconstituted cell line. This finding is in line with the reduced presence of mature 18S rRNA in TTD cells. To confirm our mass spectrometry data, we picked three ribosomal proteins of the big and small ribosomal subunit and analyzed their protein level in whole-cell lysate and in purified ribosomes. We were able to visualize a relative underrepresentation of proteins of the small ribosomal subunit in isolated ribosomes (Figure 4A right panel, Supplement S6C–E).

Final maturation of the 18S rRNA by cleavage of the 18S-E occurs in the cytoplasm after RPS15-mediated export of the pre-40S particle from the nucleoplasm (26). As RPS15 was found to be underrepresented in isolated TTD ribosomes, we asked the question if the export of pre-40S particles might be disturbed. Fluorescence-*in-situ* hybridization (FISH) was used to visualize the 18S-E in control and affected cells. As depicted in Figure 4B there is nucleoplasmic ITS1 staining in TTD cells traceable, that indicates, in conjunction with the reduced RPS15 content, a maturation defect of the 40S ribosomal subunit. To investigate if this maturation defect is mirrored in the distribution of cytoplasmic ribosomal subunits, polysomal profiling was performed. This method allows a ‘snapshot’ of ribosomal composition and subunit abundance and revealed that TFIIEβ mutation reduces the cytoplasmic content of the 40S ribosomal subunit as shown in Figure 4C.

Sucrose gradient centrifugation was employed to isolate ribosomes to assess the unfolding stability of ribosomal proteins. The purified ribosomes were incubated with the chaotropic reagent urea and the subsequently exposed hydrophobic residues of unfolded ribosomal proteins were quantified by the fluorophore BisANS. The stability of proteins against unfolding by urea is a hallmark of long-lived species (27–28) and is considered as an indicator for translational accuracy (29). After urea challenge, ribosomal preparations of both TTD cell lines depict increased amounts of unfolded ribosomal proteins (Figure 4D) indicating a qualitative disturbance of the corresponding ribosomes. These results indicate that the amount of misfolded proteins in ribosomal preparations of TFIIEβ mutant cells is higher than in reconstituted and control cells.

### TFIIEβ mutation results in a disturbed translational fidelity, protein homeostasis and cell growth

**Figure 5.**
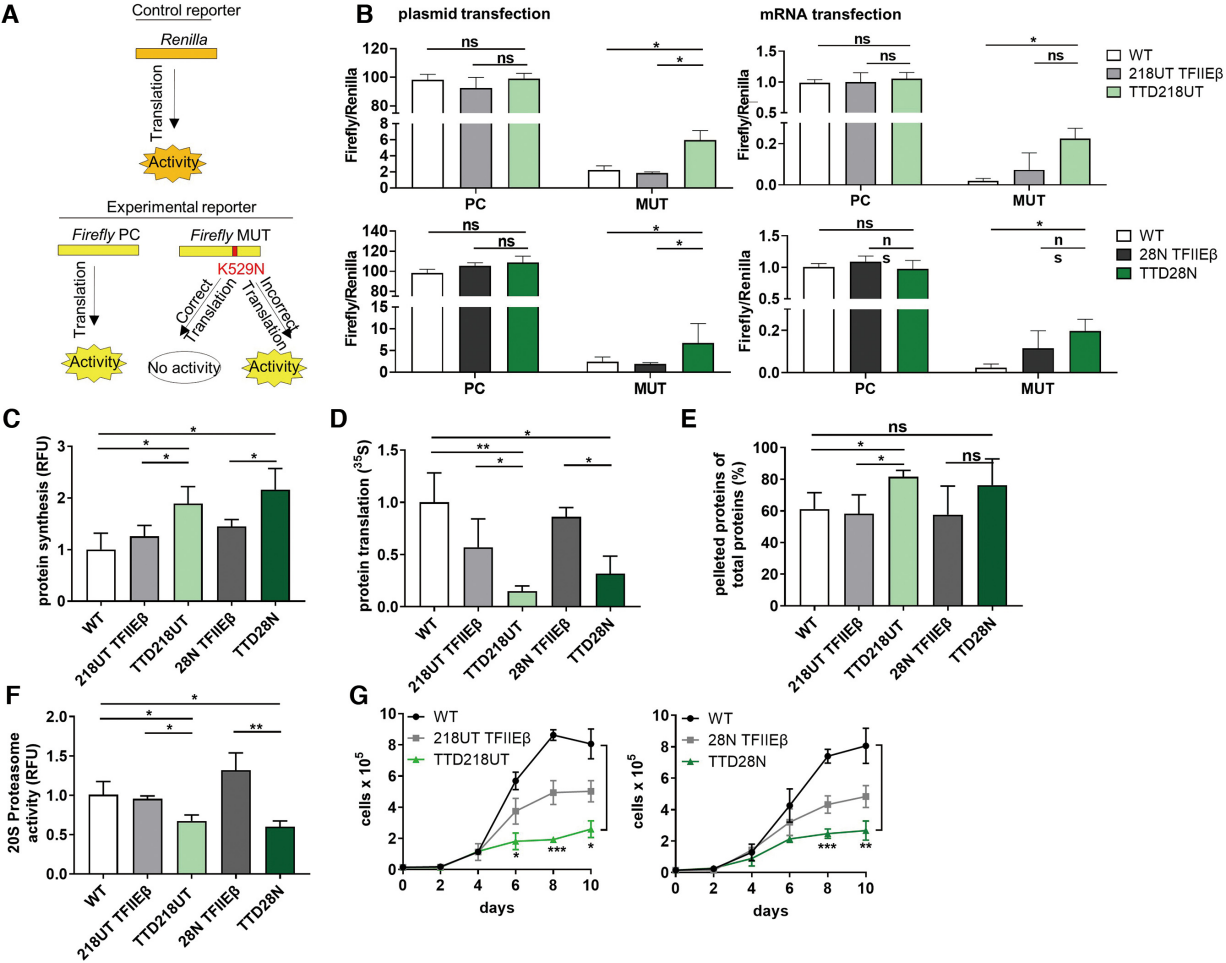
Loss of protein homeostasis in TTD cells. (**A**) Luciferase-based translation fidelity assay includes a control reporter *Renilla* and two *Firefly* reporters containing either no mutation (PC) or a point mutation K529N inactivating the *Firefly* (MUT). With correct translation, the point mutation is translated and *Firefly* is inactive, whereas with inaccurate translation the luminescence of the active *Firefly* is detected. The *Firefly* luminescence was normalized to the *Renilla* luminescence. (**B**) Left: To all cells, plasmids coding for *Renilla* were co-transfected via electroporation with either plasmids coding for *Firefly* NC or *Firefly* MUT. Translation fidelity assay with plasmids shows increased translational infidelity in TTD cells. Right: Plasmids coding for *Renilla*/*Firefly* PC or *Renilla*/*Firefly* MUT were transcribed by T7 polymerase and resulting mRNAs were transfected with lipofectamine to all cells. Translation fidelity assay with mRNAs indicates increased translational infidelity in TTD cells. (**C**) 5 FAM-Azide detection of OPP-labeled proteins indicates the rate of protein synthesis initiation in WT, reconstituted and TTD cells. TTD cells show increased translation initiation. (**D**) Analysis of total protein translation by using ^35^S metabolic labeling shows decreased protein synthesis in TTD cells compared to WT and reconstituted cells. (**E**) Heat sensitivity analysis of cytoplasmic extract in WT, reconstituted and TTD cells show increased percentage of pelleted proteins in TTD cells after heat treatment for 15 min at 99°C. (**F**) Detection of cleaved 20S specific substrate, SUC-LLVY-AMC, indicating the 20S proteasome activity in WT, reconstituted and TTD cells. Reduced proteasome activity was observed in TTD cells. (**G**) Proliferation kinetics of TTD cells compared to WT and reconstituted cells indicate reduced cell proliferation in TTD cells. Data are represented as mean ± SD of at least three independent experiments. ns *P* > 0.05, * *P* ≤ 0.05, ** *P* ≤ 0.01, *** *P* ≤ 0.001.

To further unravel the functional base of the elevated load of misfolded proteins in ribosomal proteins, we assessed the error rate of the translation process itself. For this purpose, a luciferase-based translation fidelity assay was used. This assay includes a control reporter *Renilla* and a *Firefly* reporter containing a point mutation K529N, which inactivates the *Firefly* luciferase (Figure 5A). The luciferase *Firefly* MUT stays inactive with correct translation of the point mutation, whereas luminescence of the *Firefly* MUT is detectable only in the case that erroneous translation occurs. Plasmids expressing these constructs were co-transfected in WT, reconstituted and TTD cells. Strikingly, both TTD cell lines show an increased luminescence indicative of a higher error rate when compared to the WT and reconstituted cells (Figure 5B left panels).To exclude that the re-activation of luciferase is due to errors in RNA polymerase II transcription, we transfected mRNA into cells. To further guarantee that *Renilla* : *Firefly* luciferase is transfected in a ratio of 1:1, plasmids expressing a fusion-protein of *Renilla* and *Firefly* were transcribed by means of a T7 kit and transfected to WT, reconstituted and TTD cells (Figure 5B right panels). As observed in the translational fidelity assay with plasmid transfection, TTD cells depict similar high luciferase activity of *Firefly* MUT after mRNA transfection. We therefore conclude, that the TFIIEβ mutation in TTD cells fundamentally affects the accuracy of protein synthesis at the ribosome leading to the enrichment of misfolded proteins in the ribosome itself.

Protein homeostasis is sustained by the balance of protein synthesis, maintenance and degradation. As our results unraveled a disturbance in ribosomal accuracy, we investigated the impact of translation on protein homeostasis in TTD cells. First, we detected the rate of translational initiation by analyzing *O*-propargylpuromycin (OPP) incorporation in the growing amino acid chain, which leads to a premature stop of translation. Interestingly, OPP incorporation revealed an increased translational initiation in TTD cells (Figure 5C). Strikingly, the overall protein synthesis, which was assessed by metabolic labeling, was found to be significantly reduced in TTD cells. (Figure 5D). These data suggest that increased ribosomal inaccuracy leads to higher but ineffective protein synthesis initiation. This notion was further supported by the finding that the cytoplasmic proteome of TTD cells is prone to heat denaturation. As shown in Figure 5E, more proteins are pelleted during centrifugation after heat-treatment, indicating an elevated load of misfolded proteins in TTD. Protein degradation analysis by measuring 20S proteasome activity revealed a markedly and significant reduction of the 20S proteasome activity in TTD cells (Figure 5F) that is in line with reduced overall protein synthesis. Finally, we speculate that disturbed protein homeostasis might also impact on cellular proliferation and compared growth properties of TTD cells with that of WT and reconstituted cells (Figure 5G). In comparison to WT cells, proliferation kinetics of TTD cells clearly revealed a reduced division rate in TTD cells when WT cells are in the exponential proliferation phase. Taken together, our data describe a severe disturbance in proteostasis as a novel pathomechanism which explains the observed reduced growth and proliferation rates in TTD cells, and which is most likely also responsible for the symptoms and clinical picture in TTD patients.

## DISCUSSION

The heterodimeric general transcription factor TFIIE recruits TFIIH to the preinitiation complex of RNA polymerase II promoters and is thus involved in gene expression of all protein-coding genes (14,30). Here we describe an unanticipated role of TFIIE which profoundly impairs ribosomal biogenesis. Our data shows enrichment of TFIIE in the nucleolus, at the site of RNA polymerase I transcription. TFIIE enrichment depends on RNA polymerase I activity, as its inhibition by different compounds clearly reduces nucleolar TFIIE staining. So far, a nucleolar localization and function of general transcription factors of RNA polymerase II were reported for TFIIH (10,31). A seminal publication indicates that nucleolar RNA polymerase II transcription is essential for proper rRNA biogenesis and processing (32). The authors showed that RNA polymerase II transcribes intergenic spacers (IGSs) of the rDNA to maintain nucleolar organization and hence rRNA synthesis and maturation. However, it is largely unresolved how RNA polymerase II transcribes at the IGSs of the rDNA, and which general factors are involved. We cannot exclude that the RNA polymerase II driven ribosomal protein expression is disturbed by mutant TFIIE and leads to a ribosomal assembly stress response as described in yeast (33). However, we here addressed the unique possibility that the nucleolar TFIIE enrichment is due to nucleolar RNA polymerase I transcription. In fact, our data unveil that the coding region of the rDNA, that is actively transcribed by RNA polymerase I, is bound by TFIIE and selective inhibition of RNA polymerase I transcription reduces TFIIE binding. These data highlight a direct involvement of TFIIE in RNA polymerase I transcription. The C-terminal end of TFIIEβ is capable of binding to DNA (34), suggesting that TFIIE may be bound to the rDNA through its small subunit TFIIEβ. The fact that the mutation of TFIIEβ weakens the rDNA gene-occupancy of RNA polymerase I, indicates that TFIIE has a direct impact on the process of ribosomal biogenesis. Further investigations will reveal, if reduced binding of the elongation factor TFIIH to the rDNA is also observed, as TFIIE recruits TFIIH in RNA polymerase II pre-initiation complex formation (35). A reduced gene-occupancy of RNA polymerase I, as shown in this study, was already described by Nonnekens *et al.* (12) for TTD and CS-causing mutations in TFIIH and CSB indicating that this might be a common pathophysiology in these childhood syndromes. Moreover, in this publication, the authors further showed, that co-transcriptional processing of the pre-rRNA is disturbed in cells and tissues from a TTD mouse model with a mutation in TFIIH. The process of RNA polymerase I transcription elongation is coupled to the processing and pre-ribosomal assembly (36,37), thus disturbances in transcription elongation might impact on the subsequent maturation steps. In our study, we demonstrate a previously unreported accumulation of processing intermediates and a reduced abundance of the 18S mature rRNA in TFIIEβ mutant cells. Polysomal profiling revealed a reduced content of cytoplasmic 40S subunit in TFIIEβ mutant cells. Isolation of cytoplasmic ribosomes and mass spectrometric analysis also unraveled an underrepresentation of the 40S subunit in the cytoplasm. This is in line with the accumulation of unprocessed 18S-E intermediate in the nucleoplasm, presumably part of pre-40S particles, as identified by FISH. These results are confirmed by northern blot analysis showing a reduced mature 18S in total cellular RNA. The knockdown of BMS1, an essential co-factor for rRNA processing is also leading to an underrepresentation of the 40S subunit in yeast (38). Maturation disturbances as a consequence of transcription elongation defects in the rRNA can also translate to ribosomal assembly defects as described in yeast (39). We compared ribosomal composition of patient cells with reconstituted cells and detected an underrepresentation of ribosomal proteins of the small ribosomal subunit. The small ribosomal subunit 40S is the translation initiating and codon-decoding subunit of the ribosome. Disturbed ribosomal composition can lead to decoding defects of the ribosome (40–42) detectable by luciferase-based translation assays. We took advantage of a well-established luciferase transfection system (29) and discovered an elevated error rate of the translation process in TTD patient cells. The reduced translational fidelity, described for the first time in TTD, has recently been reported in CS-patient derived fibroblasts (17). We here found translational infidelity followed by a loss of proteostasis and disturbed protein synthesis in TTD, indicating that TTD and CS share distinct aspects in their pathophysiology and this may be a more general mechanism in both childhood syndromes. In this regard, both are caused by mutations in the same subunits of the transcription/DNA repair factor TFIIH. Additionally, these similarities might suggest, that the loss of proteostasis in CS might not origin from DNA damage, as a comparable defect is here shown for TTD cells which do not have any deficiencies in Nucleotide Excision Repair (13).

The loss of proteostasis is also evident by the elevation of heat-sensitive proteins in patient cells. Heat-stability of the proteome is a hallmark of long-living species and characterizes the longest-living animal, *Arctica islandica* (27). Elevated heat sensitivity indicates that a detectable proportion of the proteome is affected by misfolding, and this most likely is caused by translational errors. In support of this, ribosomal proteins display an elevated unfolding by urea in comparison to control ribosomes, indicating that the ribosomes themselves are affected by misfolded proteins likely due to an error-prone translation process. Our findings re-vitalize the error catastrophe theory of aging (43). In his theory, Orgel postulates that elevated translational infidelity perpetuates and enhances itself by the accumulation of errors in the amino acid sequence of ribosomal proteins that have an influence on translational fidelity.

The reduced translational fidelity at the ribosome in TFIIEβ mutant cells might be the cause of the observed loss of proteostasis. Interestingly, we detected an increase in the initiation of protein synthesis in TTD cells, while total protein synthesis is significantly reduced. This supports the assumption that the translation process is inefficient and error-prone in TFIIEβ cells. TTD is mainly regarded as a transcriptional syndrome, as 50% of the cases do not display DNA-repair disturbances (44). We here extend this classification and highlight a possible contribution of protein translation disturbances in the pathophysiology of TTD. Recently, mutations in aminoacyl-tRNA synthetases were identified to cause TTD (15,16) implying that a disturbed translation process, as shown in this study, might be causal for the developmental delay and neurodegeneration observed in TTD. This assumption is based on the hypothesis that only disturbances in a common cellular signaling pathway that is affected by all genes in this genotypical heterogenous disease can explain the phenotype. Here we provide first evidence, that the translation process at the ribosomes might be this common pathway. If also mutations in the TTD-causing genes TTDN1 (MPLKIP) and RNF113A are impacting the translation process awaits further analysis.

## DATA AVAILABILITY

All unique/stable reagents generated in this study are available from the Lead Contact with a completed Materials Transfer Agreement. Proteomics raw data, MaxQuant search results and the used protein sequence database have been deposited with the ProteomeXchange Consortium via the PRIDE partner repository (https://www.ebi.ac.uk/pride/) and can be accessed using the data set identifier PXD023451.

## Supplementary Material

gkab866_Supplemental_File
