# Supplementary material for: Nucleolar TFIIE plays a role in ribosomal biogenesis and performance
Source: Nucleic Acids Res. 2021 Sep 28;49(19):11197–210. doi: 10.1093/nar/gkab866 (PMC8565312; doi:10.1093/nar/gkab866)
Supplement: gkab866_Supplemental_File [file gkab866_supplemental_file.pdf]

**Supplementary Material: Tables S**

**Table S1.** Antibodies of TFIIE, TFIIH, RNA polymerase I transcription machinery, Nucleolus and Ribosomal Proteins are listed below.

| Antibody | Source | Identifier | Application |
|---|---|---|---|
| **TFIIE** | | | |
| rabbit polyclonal TFIIEα | Abcam | ab28177 | ChIP |
| mouse monoclonal TFIIEα | Santa Cruz | sc-374014 (B-7) | IF |
| rabbit polyclonal TFIIEβ | GeneTex | GTX105029 | WB |
| rabbit polyclonal TFIIEβ | GeneTex | GTX109005 | WB |
| rabbit monoclonal TFIIEβ | Abcam | ab187143 | ChIP, IF |
| **TFIIH** | | | |
| mouse monoclonal p62 | Santa Cruz | sc-25329 (H-10) | WB |
| mouse monoclonal XPD | Abcam | ab54676 | WB |
| mouse monoclonal cdk7 | Santa Cruz | sc-7344 (C-4) | WB |
| **RNA Polymerase I transcription machinery / Nucleolus** | | | |
| mouse monoclonal UBF | Santa Cruz | sc-13125 (F-9) | WB |
| mouse monoclonal TAF I p110 | Santa Cruz | sc-374551 (C-10) | WB |
| mouse monoclonal Rrn3 | Santa Cruz | sc-390464 (D-9) | WB |
| rabbit polyclonal RPA194 | Santa Cruz | sc-28714 (H-300) | WB |
| mouse monoclonal RPA194 | Santa Cruz | sc-46699 (F-6) | WB, IF |
| mouse monoclonal RPA135 | Santa Cruz | sc-293272 (4H6) | WB |
| RPA135 | homemade | | WB, ChIP |
| rabbit monoclonal Fibrillarin | Abcam | ab166630 | IF |
| mouse monoclonal Fibrillarin | Abcam | ab4566 | IF |
| rabbit monoclonal Nucleolin | Cell Signaling | 14574S | IF |
| mouse monoclonal Nucleolin | Cell Signaling | 87792S | IF |
| **Ribosomal proteins** | | | |
| mouse monoclonal RPL4 | Santa Cruz | sc-100838 (RQ-7) | WB |
| mouse monoclonal RPL5 | Santa Cruz | ab137617 | WB |
| Mouse monoclonal RPL 9 | Santa Cruz | sc-100828 | WB |
| mouse monoclonal RPL17 | Santa Cruz | sc-515904 (C-8) | WB |
| rsabbit monoclonal RPS15 | Abcam | ab157193 | WB |
| rabbit monoclonal RPS20 | Abcam | ab133776 | WB |
| mouse monoclonal RPSA | Santa Cruz | sc-101517 (16) | WB |

**Table S2.** Sequences (5' to 3') of primers used to amplify rDNA regions are listed below.

| **Primer** | **Forward** | **Reverse** | **Position (bp)** |
|---|---|---|---|
| rDNA1 | ACCTAGCGGTCACTGTTACTC | TCAAAGTGGCGATTTCCTAG | -4957 |
| rDNA2 | TCTGTCTCTGCGTGGATTC | AGGGAGGGAGAAAGAACAC | -1200 |
| rDNA3 / promoter region | CTGCGATGGTGGCGTTTTTG | ACAGCGTGTCAGCAATAACC | -85 |
| rDNA4 / 47S | TGTCAGGCGTTCTCGTCTC | AGCACGACGTCACCACATC | 356 |
| rDNA5 | GCTCTGCCTCGGAAGGAAG | CTGCGGTACGAGGAAACAC | 1241 |
| rDNA6 | GACGACCCATTCGAACGTCT | CTCTCCGGAATCGAACCCTG | 4032 |
| rDNA7 / 18S | CGATGCGGCGGCGTTATTCC | GAACGGCCATGCACCACCAC | 4796 |
| rDNA8 | CGTTGAACCCCATTCGTG | TATGACCCGCACTTACTCG | 5281 |
| rDNA9 / ITS1 | GCGGAGGTTTAAAGACCCCTTGG | GTCGGAAGGTTTCACACCACGG | 6164 |
| rDNA10 | TCGTGCGTCGATGAAGAACGCAG | ATTGATCGGCAAGCGACGCTCAG | 6671 |
| rDNA11/ 28S | AGTCGGGTTGCTTGGGAATGC | CCCTTACGGTACTTGTTGACT | 8260 |
| rDNA12 / 28S beginning | CATCAGACCCCAGAAAAGG | TGATTCGGCAGGTGAGTTG | 9900 |
| rDNA13 | GGCATGTTGGAACAATGTAGG | CCTTAGAGCCAATCCTTATCC | 10781 |
| rDNA14 / 28S middle | CGCCTAGCAGCCGACTTAG | GTTACTCCCGCCGTTTACCC | 11590 |
| rDNA15/ 28SETS | ACCTGGCGCTAAACCATTCGT | GGACAAACCCTTGTGTCGAGG | 12901 |
| rDNA16 / IGS | CCACAGGTCGAGGCTTCGGT | GGCGCTCTCTGCGTCTCACT | 19086 |

**Supplementary Material: Figure S Legends**

**Figure S1. Co-localization studies of TFIIE, TFIIH and TFIIF within the nucleolus using STED microscopy. (A)** STED images of TFIIH (left) and TFIIF (right) with nucleolus marker RNAP1, Fibrillarin and Nucleolin showing strong co-localization of TFIIH with all nucleolus marker and slight co-localization of TFIIF with RNAP1. Scale bar 5 µm. **(B)** STED images of TFIIEβ after specific inhibition of RNA polymerase I transcription by BMH-21 indicating re-organization of RNAP1 and Fibrillarin to nuclear caps and re-distribution of TFIIEβ enrichment from the nucleolus. Scale bar 5 µm. **(C)** Confocal immunofluorescence microscopy (100X) of WT after ActinomycinD treatment indicate re-distribution of TFIIEβ from the nucleolar enrichment and an organization of RNAP1 to nucleolar caps (arrows). Scale bar 5 µm.

**Figure S2. Quantification of co-localization studies. (A-B)** Plot Profile of STED images presented in Figure 1A and Figure S1A. Images were analyzed with ImageJ by drawing a line through the nucleolus and measuring the gray value of TFIIEβ (A), TFIIH (B, left) and TFIIF (B, right) with RNAP1, Fibrillarin and Nucleolin. Gray value peaks of TFIIEβ and TFIIH resemble the peaks of RNAP1, Fibrillarin and Nucleolin, while the peaks of TFIIF are more flat indicating less enrichment of TFIIF to the nucleolus compared to TFIIEβ and TFIIH. **(C)** Plot profile of STED images in Figure S1B showing a distinguish plot profile of TFIIEβ and the nucleolus marker RNAP1 and Fibrillarin after BMH-21 treatment. **(D)** Quantification of IF performed in Figure 1D showing no significant changes of TFIIEα localization after inhibition of RNA polymerase I transcription by CX5461.

**Figure S3. Binding of TFIIEβ to the same rDNA molecule as RNAP1 and UBF at 28S coding region of the rDNA. (A)** Quantitative qPCR analysis of ChIP performed in Figure 2A showing no significant binding of TFIIEβ to the Promotor, 18S coding region, but significant binding to the beginning of the 28S coding region. IGS serves as a negative control. **(B)** Semi-quantitative PCR analysis and of ChIP and sequential ChIP indicating TFIIEβ binds to the same rDNA molecule as RNAP1 and UBF at the 28S coding region. Quantitative qPCR analysis of ChIP (C) and sequential ChIP (D) performed in (B) showing significant binding of TFIIEβ to the same rDNA molecule as UBF at the 28S coding region. **(E)** Full images of Co-immunipercipitation performed in Figure 2E. Data are represented as mean ± SD. ns $p > 0.05$, * $p \leq 0.05$, ** $p \leq 0.01$, *** $p \leq 0.001$, **** $p \leq 0.0001$ of three independent experiments.

**Figure S4. Reduced TFIIE protein level in TTD cell line. (A)** Sequence analysis of TTD218UT (left) and TTD28N (right) compared to WT shows a homozygous mutation (c.559C>A [p.Asp187Tyr]) in both TTD cell lines. **(B)** Immunofluorescence microscopy (100X) of WT cells reveal co-localization of TFIIE and nucleolus marker including Fibrillarin and Nucleolin in WT and reconstituted cells. Reduced fluorescence signals of both TFIIE subunits were observed in TTD cells. **(C)** Western Blot analysis indicates reduced protein abundance of both TFIIE subunits in TTD cells compared to WT and reconstituted cells, whereas the protein abundance of TFIIH is unaffected in TFIIEβ-mutated cells. **(D-E)** Quantification of western blot analysis in Figure S4 C indicate significant reduced protein level of both TFIIE subunits and wild-type protein level of TFIIH.

**Figure S5. Disturbed rRNA synthesis in TTD cells. (A) Left:** Nuclear Run-On assay shows level of freshly transcribed pre-rRNA in WT, reconstituted and TTD cells. Primer used for Nuclear Run-on analysis are indicated by arrowheads and numbers in the schem of the rRNA subunit. Exact primer sequence are given in Table S2. Nuclear Run-on assay detects in TTD cells significant reduced level of freshly transcribed pre-rRNA starting from 5.8S coding region to 28S coding region of the rDNA indicating a disturbance in the elongation of RNA polymerase I transcription in TTD cells. **Right:** Nuclear Run-on assay in WT, reconstituted and TTD cells detecting mRNA of actin shows no significant differences. **(B)** Quantification of Northern Blot performed in Figure 3C indicating significantly increased mature 5S rRNA level in TTD218UT and increased tendency in TTD28N.

**Figure S6. Disturbed rRNA processing and ribosomal composition in TTD cells. (A)** Full images of northern blot analysis. Membranes were probed with ITS1 (red), stripped, and re-probed with ITS2 (purple). **(B)** Quantification of 18S-E/47S ratio in WT, reconstituted and TTD cells indicating increased 18S-E level in TTD cells. **(C)** Volcano plot shows a non-significant decreased amount of ribosomal protein of the small 40S ribosome subunit (RPS) and a non-significant increased amount of ribosome protein of the big 60S ribosome subunit (RPL) in TTD cells compared to reconstituted cells. **(D)** Western blot analysis of whole cell lysate and isolated ribosome in WT, reconstituted 218UT TFIIEβ cells and TTD218UT cells. **(E)** Quantification of Western Blots of three ribosomal preparations and selected ribosomal proteins point towards a tendency of over- and underrepresented proteins in lysates and ribosomal preparations. **(F)** Quantification of FISH analysis performed in Figure 4B showing no significant differences in the CTCF of U2 in TTD cells.

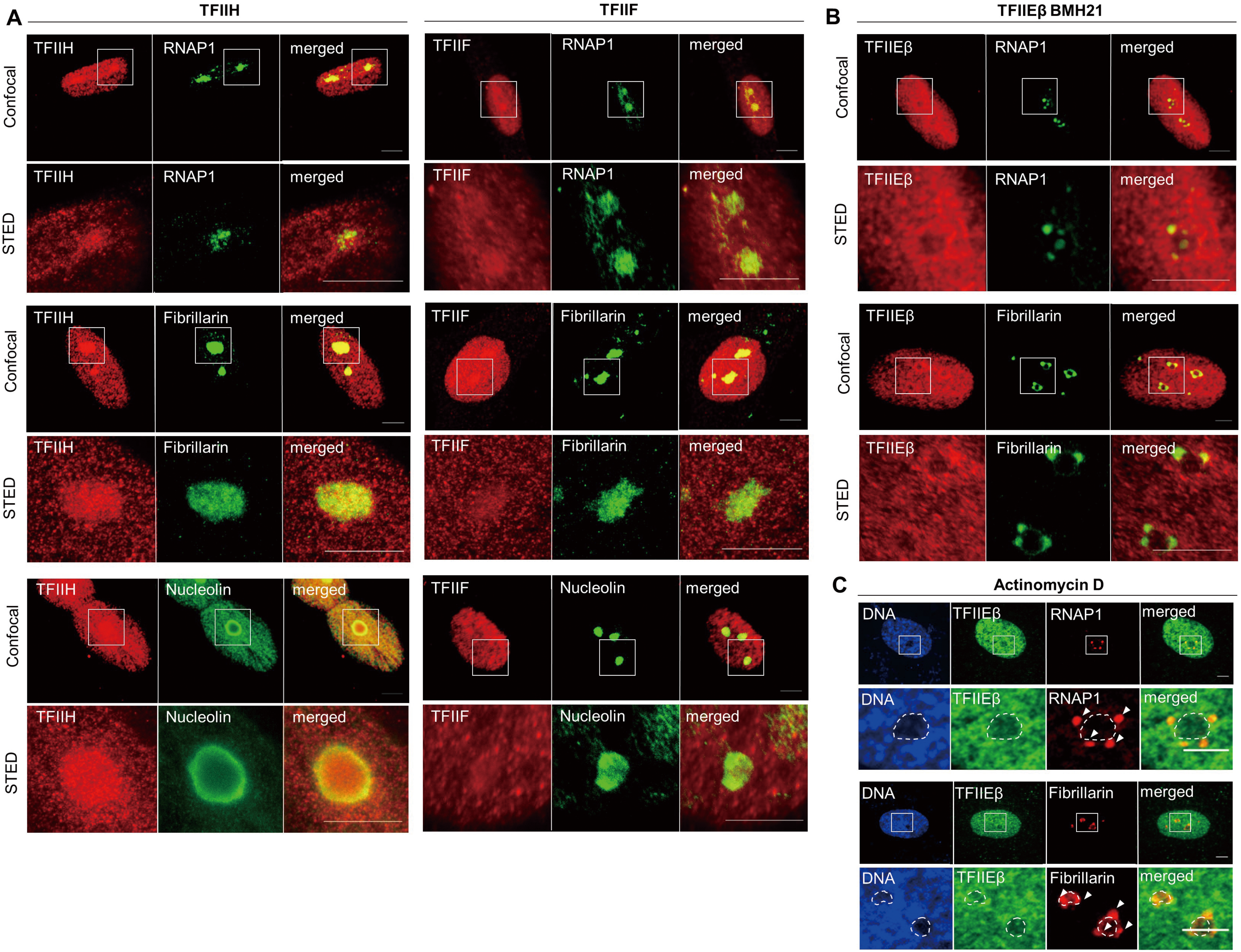
A
TFIIH
TFIIF
B
TFIIEβ BMH21
Confocal
STED
TFIIH
RNAP1
merged
TFIIF
Fibrillarin
Nucleolin
TFIIEβ
C
Actinomycin D
DNA

Figure S1

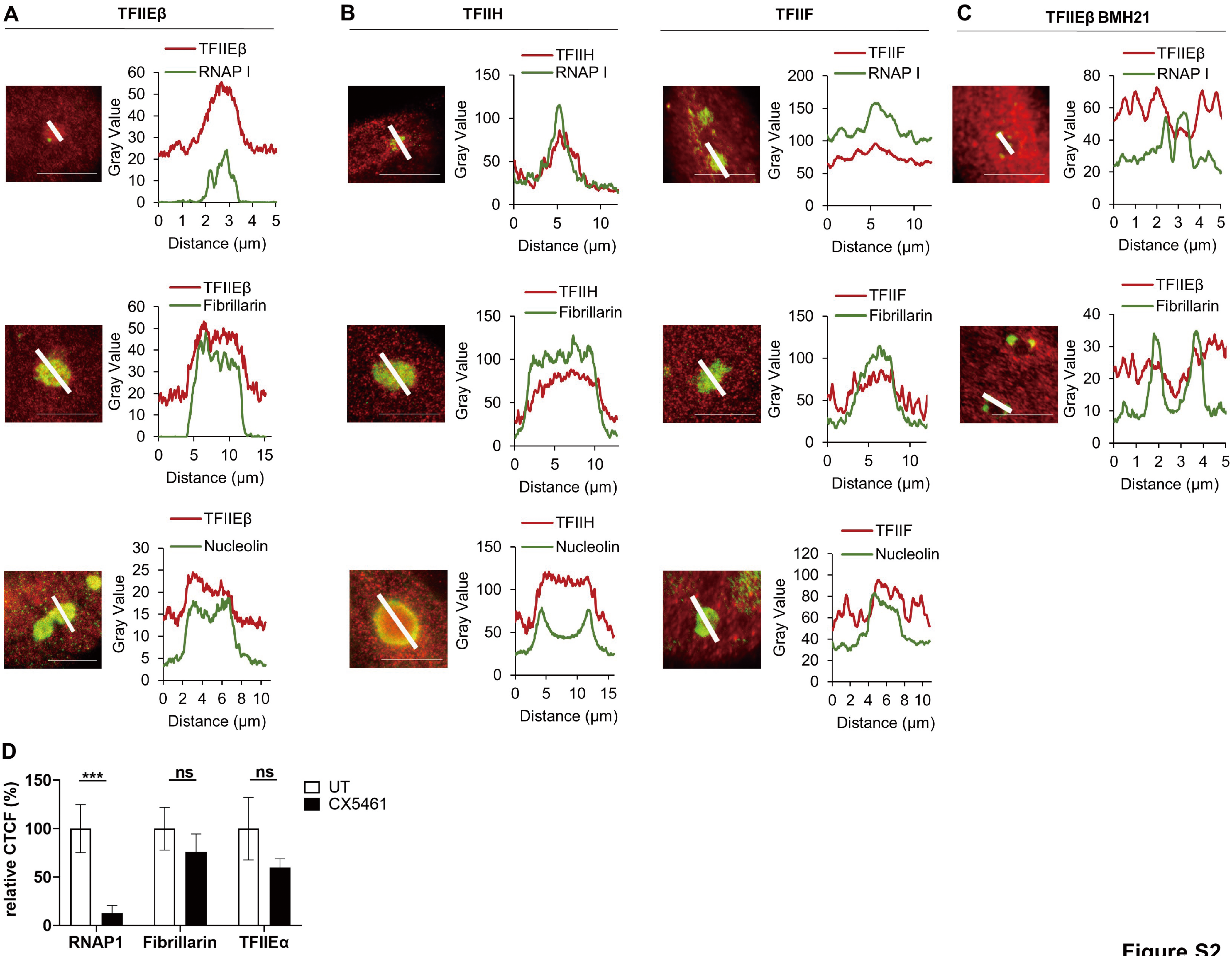
A
TFIIEβ
B
TFIIH
TFIIF
C
TFIIEβ BMH21
TFIIEβ
RNAP I
Fibrillarin
Nucleolin
TFIIH
TFIIF
Gray Value
Distance (μm)
D
relative CTCF (%)
***
ns
ns
UT
CX5461
RNAP1
Fibrillarin
TFIIEα

Figure S2

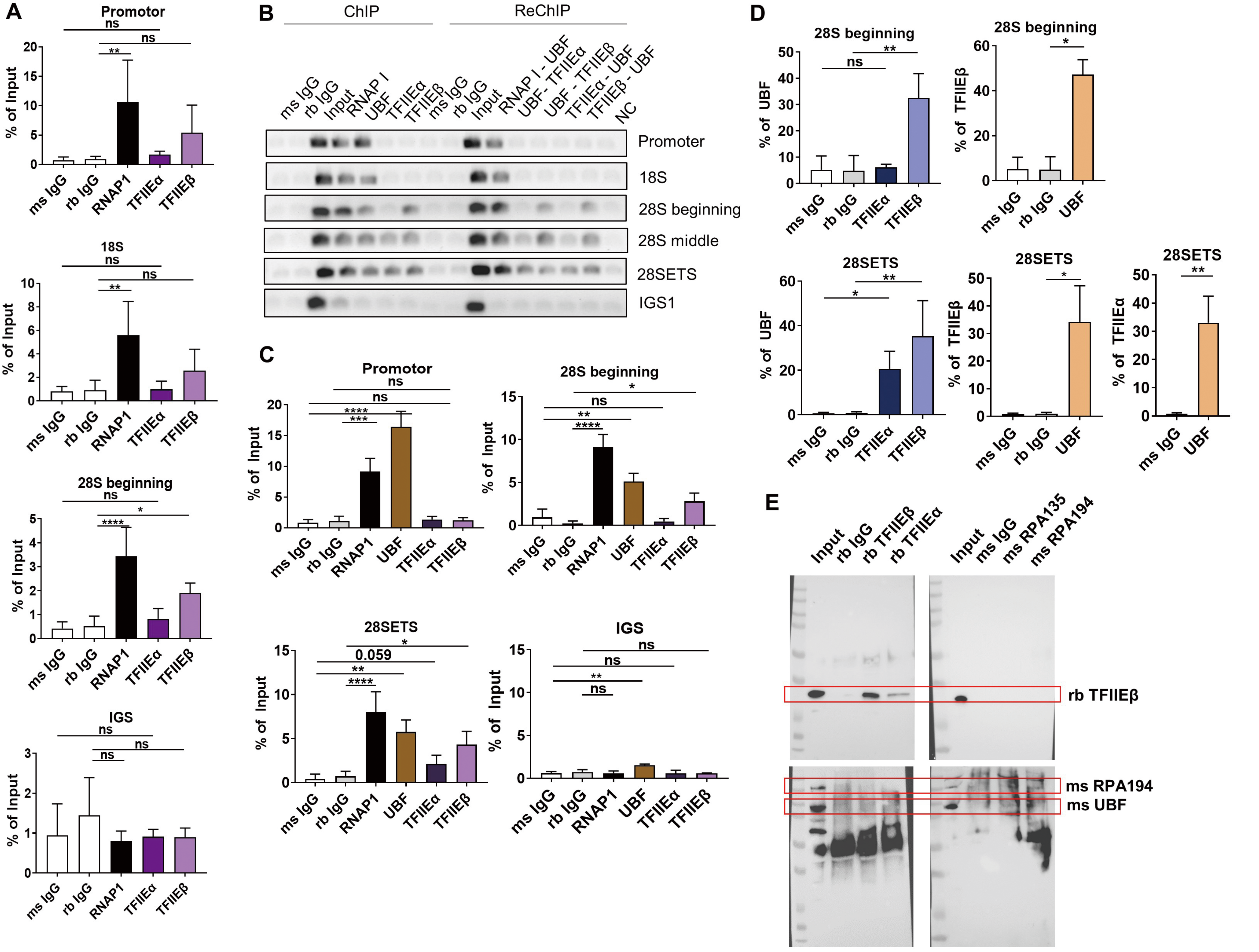

A
Promotor
18S
28S beginning
IGS
% of Input
ms IgG
rb IgG
RNAP1
TFIIEα
TFIIEβ
ns
B
ChIP
ReChIP
ms IgG
rb IgG
Input
RNAP I
UBF
TFIIEα
TFIIEβ
RNAP I - UBF
UBF - TFIIEα
UBF - TFIIEβ
TFIIEα - UBF
TFIIEβ - UBF
NC
Promoter
18S
28S beginning
28S middle
28SETS
IGS1
C
Promotor
28S beginning
28SETS
IGS
0.059
D
28S beginning
28SETS
% of UBF
% of TFIIEβ
% of TFIIEα
E
Input
rb IgG
rb TFIIEβ
rb TFIIEα
ms IgG
ms RPA135
ms RPA194
rb TFIIEβ
ms RPA194
ms UBF


Figure S3

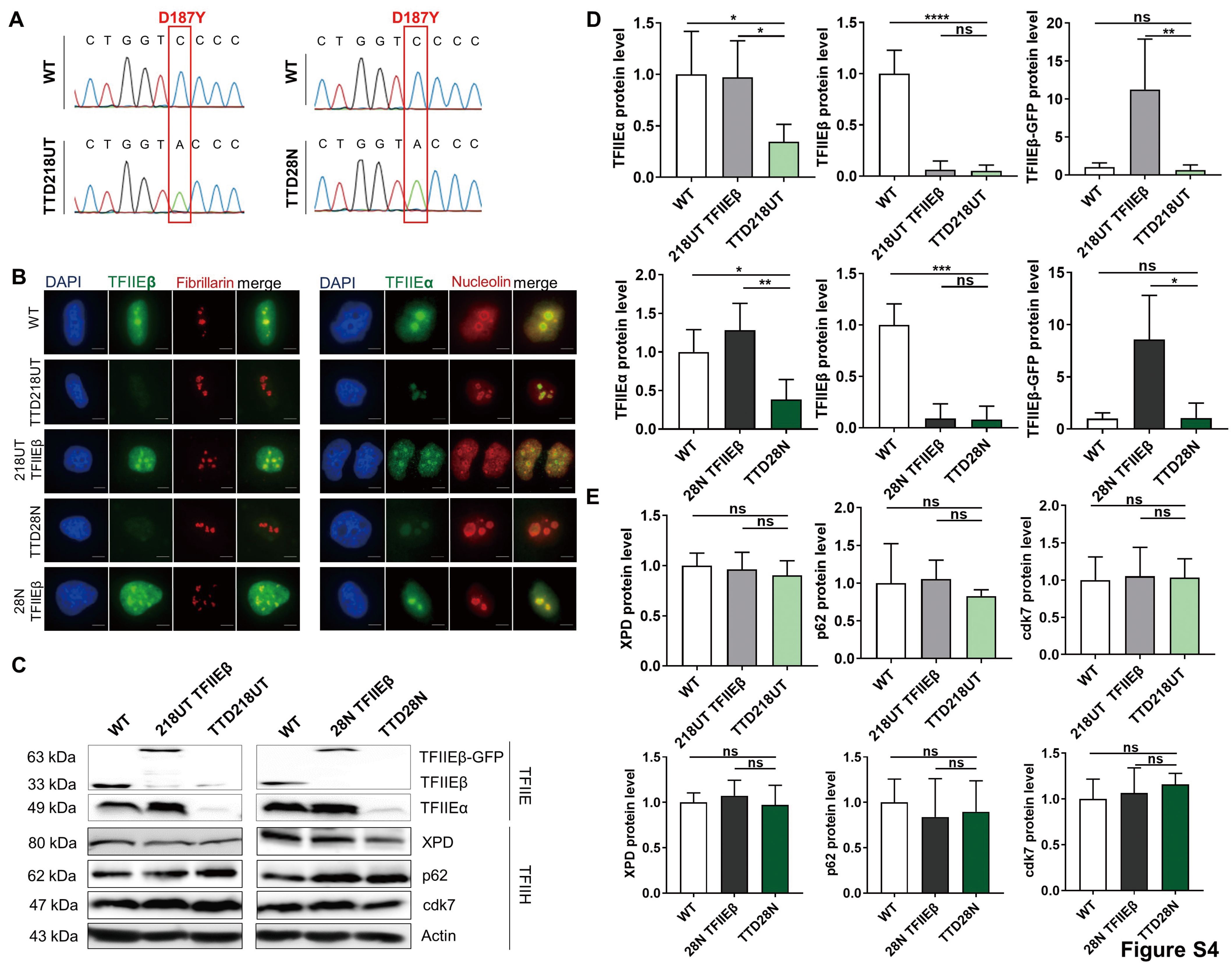
A
D187Y
WT
TTD218UT
TTD28N
C T G G T C C C C
C T G G T A C C C
B
DAPI
TFIIEβ
Fibrillarin
merge
TFIIEα
Nucleolin
WT
TTD218UT
218UT TFIIEβ
TTD28N
28N TFIIEβ
C
WT
218UT TFIIEβ
TTD218UT
28N TFIIEβ
TTD28N
63 kDa
33 kDa
49 kDa
80 kDa
62 kDa
47 kDa
43 kDa
TFIIEβ-GFP
TFIIEβ
TFIIEα
XPD
p62
cdk7
Actin
TFIIE
TFIIH
D
TFIIEα protein level
TFIIEβ protein level
TFIIEβ-GFP protein level
E
XPD protein level
p62 protein level
cdk7 protein level
ns
Figure S4

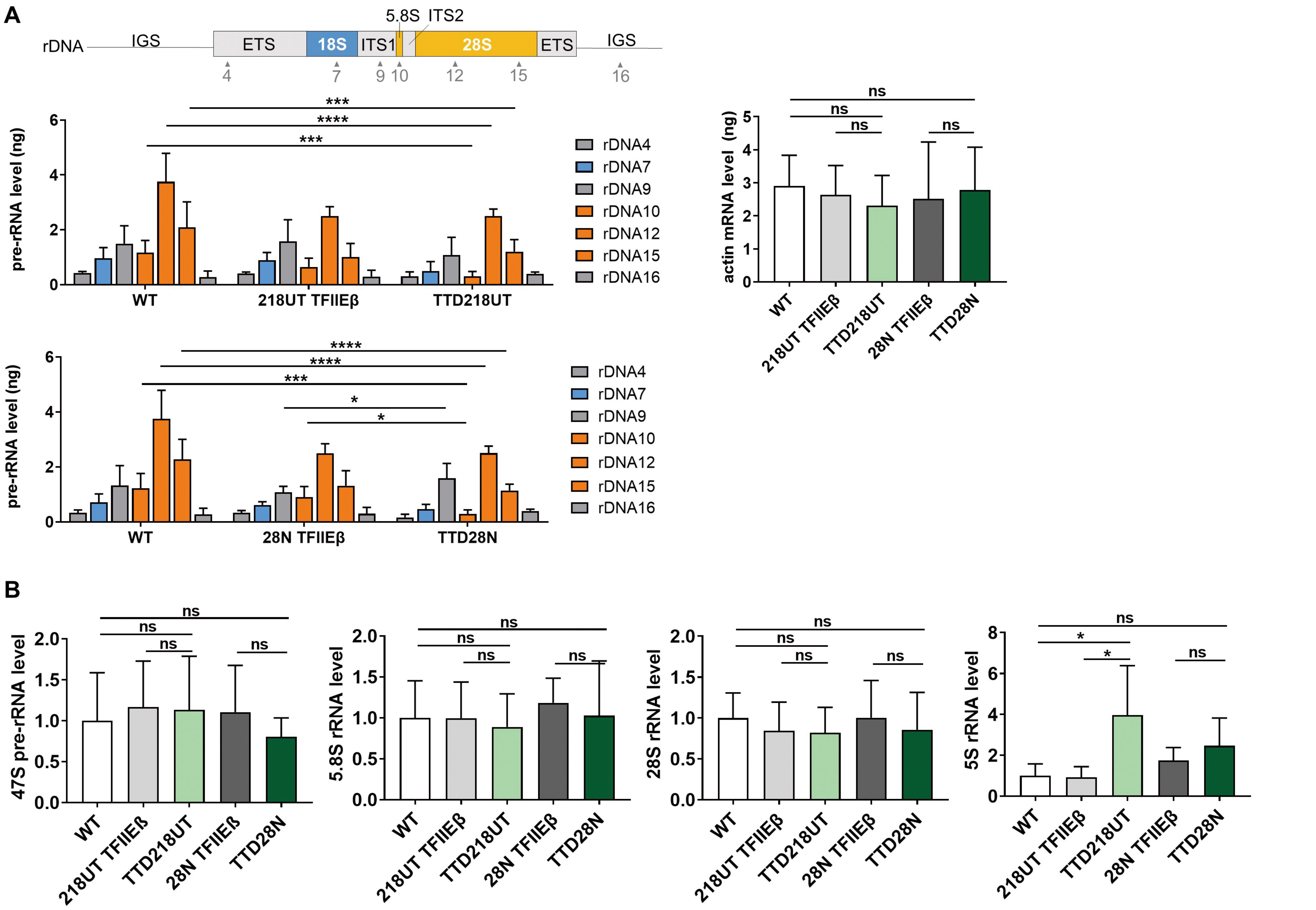
A
rDNA
IGS
ETS
18S
ITS1
5.8S
ITS2
28S
ETS
IGS
4
7
9
10
12
15
16
pre-rRNA level (ng)
rDNA4
rDNA7
rDNA9
rDNA10
rDNA12
rDNA15
rDNA16
WT
218UT TFIIEβ
TTD218UT
***
****
***
actin mRNA level (ng)
ns
WT
218UT TFIIEβ
TTD218UT
28N TFIIEβ
TTD28N
pre-rRNA level (ng)
WT
28N TFIIEβ
TTD28N
****
****
***
*
*
B
47S pre-rRNA level
5.8S rRNA level
28S rRNA level
5S rRNA level
ns
*
WT
218UT TFIIEß
TTD218UT
28N TFIIEß
TTD28N

Figure S5

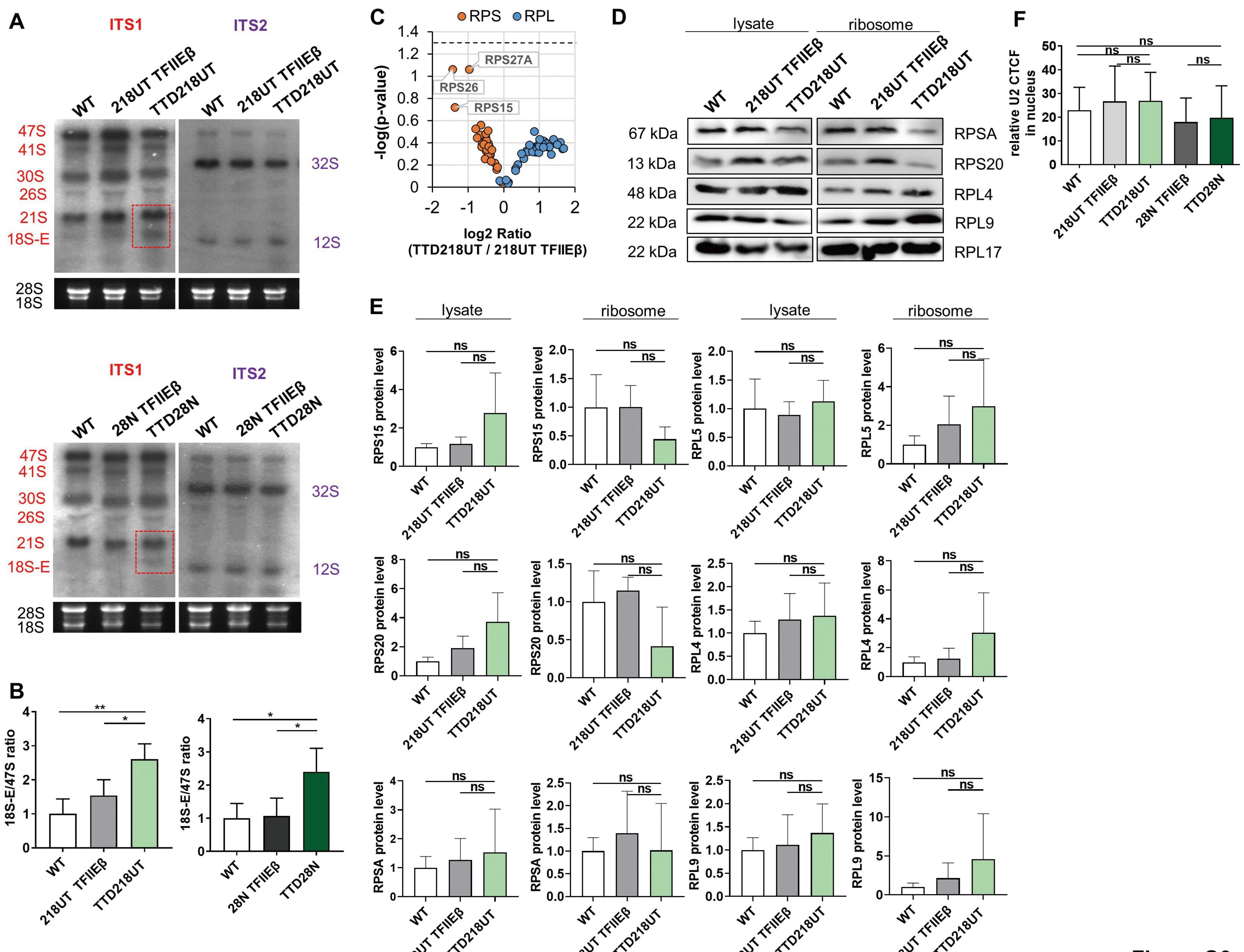
A
ITS1
ITS2
WT
218UT TFIIEβ
TTD218UT
47S
41S
30S
26S
21S
18S-E
32S
12S
28S
18S
28N TFIIEβ
TTD28N
B
18S-E/47S ratio
C
RPS
RPL
-log(p-value)
RPS27A
RPS26
RPS15
log2 Ratio
(TTD218UT / 218UT TFIIEβ)
D
lysate
ribosome
67 kDa
13 kDa
48 kDa
22 kDa
RPSA
RPS20
RPL4
RPL9
RPL17
E
RPS15 protein level
RPL5 protein level
RPS20 protein level
RPL4 protein level
RPSA protein level
RPL9 protein level
ns
F
relative U2 CTCF in nucleus

Figure S6
